# Supplementary material for: Chemogenomics and orthology‐based design of antibiotic combination therapies
Source: Mol Syst Biol. 2016 May 23;12(5):872. doi: 10.15252/msb.20156777 (PMC5289223; doi:10.15252/msb.20156777)
Supplement: Supplementary file 1 — Appendix [file MSB-12-872-s001.docx]

**Appendix for**

**Chemogenomics and Orthology-based Design of Antibiotic Combination Therapies**

Sriram Chandrasekaran, Melike Cokol-Cakmak, Nil Sahin, Kaan Yilancioglu, Hilal Kazan, James J. Collins and Murat Cokol

Appendix Supplementary Methods: 2

Appendix Figures: 6

Appendix Figure S1: Growth Data for E. coli grown with different antibiotic combinations (both training and test set). 9

Appendix Figure S2: Concordance between the interaction scores from the Loewe’s Additivity Model and Bliss Interaction Model. 10

Appendix Figure S3: Benchmarking experimental data (this study) with Yeh et al drug interaction data set. 11

Appendix Figure S4: INDIGO accurately predicts novel drug interactions (ROC curves). 12

Appendix Figure S5. Comparison with O2M 13

Appendix Figure S6: Effect of changing the threshold for synergy and antagonism 15

Appendix Figure S7. Estimating statistical significance of predictions by INDIGO by comparing to a random model of drug interaction prediction. 16

Appendix Figure S8. Effect of removing the outlier hydrogen peroxide on model predictions. 17

Appendix Figure S9: Cross validation analysis across individual drugs (Leave one drug out cross validation). 18

Appendix Figure S10. ROC curves for Ten-fold (orange curve) and Leave-one-drug-out (lavender curve) cross validation. 19

Appendix Figure S11. Controls and cross validation analyses to test INDIGO 20

Appendix Figure S12. Controls and cross validation analyses to test INDIGO (AUC values). 21

Appendix Figure S13: Growth Data for S. aureus grown with different antibiotic combinations. 23

Appendix Figure S14: A. Scatter plot of experimentally measured interaction scores of the two species. 24

Appendix Figure S15. INDIGO accurately predicts drug interactions in an independent study. 25

Appendix Figure S16: Positive control for running INDIGO 26

Appendix Tables: 27

Appendix Table S1: Predicted and measured interaction score for new drug interaction pairs in the test set by INDIGO. 29

Appendix Table S2: Antibiotics used and their MIC in *S. aureus* 30

Appendix Table S3: Prioritizing interactions for testing in S. aureus 31

Appendix Table S4: Drug Interaction scores in S. aureus. 33

Appendix Table S5: Drug Interaction scores in M. tuberculosis. 34

Appendix Table S6: Effect of Dose of Chemogenomics Profiles on INDIGO Predictions. 35

References: 35

# Appendix Supplementary Methods:

**Bliss interaction score:** We also quantified the dose-specific interactions based on the excess over Bliss metric. We used the Bliss score due to enable comparison with other drug combination data (Yeh et al, 2006). The Bliss ε score is defined as

ε = W_xy_ – W_x_*W_y_

where W_x_ and W_y_ are the normalized growth rates caused by the addition of each of the single antibiotics, and W_xy_ is the normalized growth rate caused by the addition of both antibiotics. The Bliss score ε estimates the deviation of the combined effect of two compounds to an independent model of their individual effects. Interactions with negative ε are synergistic, while those that are positive are antagonistic. The growth level in each condition was defined as the area under the growth curve normalized by the area under the curve in the ‘no drug’ condition.

**Comparison with existing drug interaction data:** We chose drugs that overlap with Yeh et al study to compare our approach to existing data sets in literature. While we have done a multi-dose study, existing studies such as Yeh et al are single-dose measurements. Among the total 1539 dose-specific measurements (171 pairs x 9 dose combinations) in our study, only 66 dose-specific measurements overlaps with Yeh et al. By using the bliss metric to evaluate our interactions and matching the dose used by Yeh et al, we found that the overall correlation between the two data sets among the 66 interactions that were shared was 0.42 (p-value = 0.0004). In addition to dose, the differences in growth media and the assay used for assessing growth, also affects the correlation between the two data sets. Among the 66 interactions, only one interaction disagreed completely i.e was synergistic in one data set and antagonistic in the other. 27 were exact matches, while the remaining 38 were predicted to be synergistic or antagonistic in one data set but predicted as non-interacting in the other. Our improved experimental setup that looks at a larger dose range (rather than single dose in Yeh et al) should better represent the drug interaction outcomes.

We also observed very strong antagonism of hydrogen peroxide with other antibiotics, not observed in a previous study (Brynildsen et al, 2013). Brynildsen et al predicted synergy between bactericidal antibiotics and gene knockouts that lead to the generation of intracellular hydrogen peroxide. Dwyer et al (Dwyer et al, 2014) recently highlighted that the levels of intracellular and extracellular hydrogen peroxide can be very different, and not reach equilibrium, due to biological constraints on hydrogen peroxide diffusion across the membrane, compartment-specific scavenging of hydrogen peroxide, and rapid Fenton chemistry destruction of intracellular hydrogen peroxide. The extracellular addition of hydrogen peroxide in our study may hence not have a similar effect as intracellular generation. At this high concentration, hydrogen peroxide has been observed to be bacteriostatic (Imlay, 2015) and hence leads to antagonism with many antibiotics, consistent with other studies (Lobritz et al, 2015; Ocampo et al, 2014).

**Random forests:** Random forests, a machine learning approach, is being increasingly applied in bioinformatics due to its nonparametric framework and its ability to run efficiently on large datasets without over-fitting. Random forests are effective in dealing with small sample size and high-dimensional data with complex data structures (Qi, 2012). The regression random forest algorithm used in this study creates an ensemble of decision trees and outputs the mean prediction of the individual trees; it also provides an automatic measure of feature importance (Strobl et al, 2007). We used the RandomForest toolbox in MATLAB.

**Chemogenomic data processing:** Chemogenomic data for *E. coli* were obtained from Nichols et al. Data were quantile-normalized using the quantilenorm function in MATLAB. Interactions with chemogenomics fitness score less than -2 (two standard deviations below the mean) were chosen to be significant and used as input to INDIGO. If multiple chemogenomic profiles with different doses matched to the same drug, we chose the chemogenomic profile whose dose best matched the MIC in our experimental drug interaction data. We found that predictive accuracy decreased when the dosage used for chemogenomics measurement and drug interactions were not matched appropriately (correlation for leave-one-drug-out cross validation – 0.42; Appendix Table S6). The predictive ability of the model can be improved by matching the dosage used in chemogenomics and drug interaction data or by analyzing across multiple doses to determine the most robust interactions.

In this study, we only used genes that lead to increased sensitivity (and not resistance) in the chemogenomics data. As a result of the Nichols et al study design, there were lot more statistically significant associations for sensitivity than resistance. 80% of the reported phenotypes were negative (gene deletion more sensitive) and 20% positive (gene deletion more resistant), consistent with other recent chemical-genetic interaction analyses in *S. cerevisiae* and *S. pombe*. Hence, only gene sensitivity profiles were used as they were more abundant and statistically significant in Nichols et al. In the future, it should be possible to include resistant genes in the INDIGO framework.

**Precision, recall and AUC calculation:** Precision was defined as the fraction of identified interactions that are true positives (TP/P); recall or sensitivity measures the true positive rate, which is the fraction of true positives interactions correctly identified; specificity measures the true negative rate. AUC values were determined by the *prefcurve* function in MATLAB, which measures sensitivity and specificity of model predictions over a range of thresholds and estimates the area under the ROC curve (AUC). The ROC curve is obtained by plotting the true positive rate against the false positive rate (1 – specificity). P-values for AUC calculations were determined by random permutation of the experimental measurement (Appendix Figure S7). Significance of individual AUC values was then estimated by comparison with the background distribution of random permutations using a t-test.

**Global analysis of drug interactions:** Drugs were grouped based on target processes and chemical class using annotation from Nichols et al (Nichols et al, 2011). To determine synergistic or antagonistic interactions between different groups, we compared the interaction scores for all drugs in each class with the background interaction score for all drug pairs. The t-score from a t-test for enrichment for synergy (negative t-score) or antagonism (positive t-score) were plotted as a heat-map (Figure 4).

**Finding genes and pathways associated with synergy or antagonism:** Genes significantly associated with synergy or antagonism were identified based on a two sampled t-test with unequal variance. Interaction profiles containing sensitivity in the gene of interest were compared with interaction profiles without sensitivity in the gene of interest. A significant difference between the two cases (p-value < 0.05) was considered to be associated with synergy or antagonism depending on the direction of difference. Genes that were strongly associated with synergy or antagonism were then subsequently used for pathway enrichment analysis. KEGG annotations for *E. coli* were downloaded using the R Bioconductor GAGE Package.

# Appendix Figures:

**
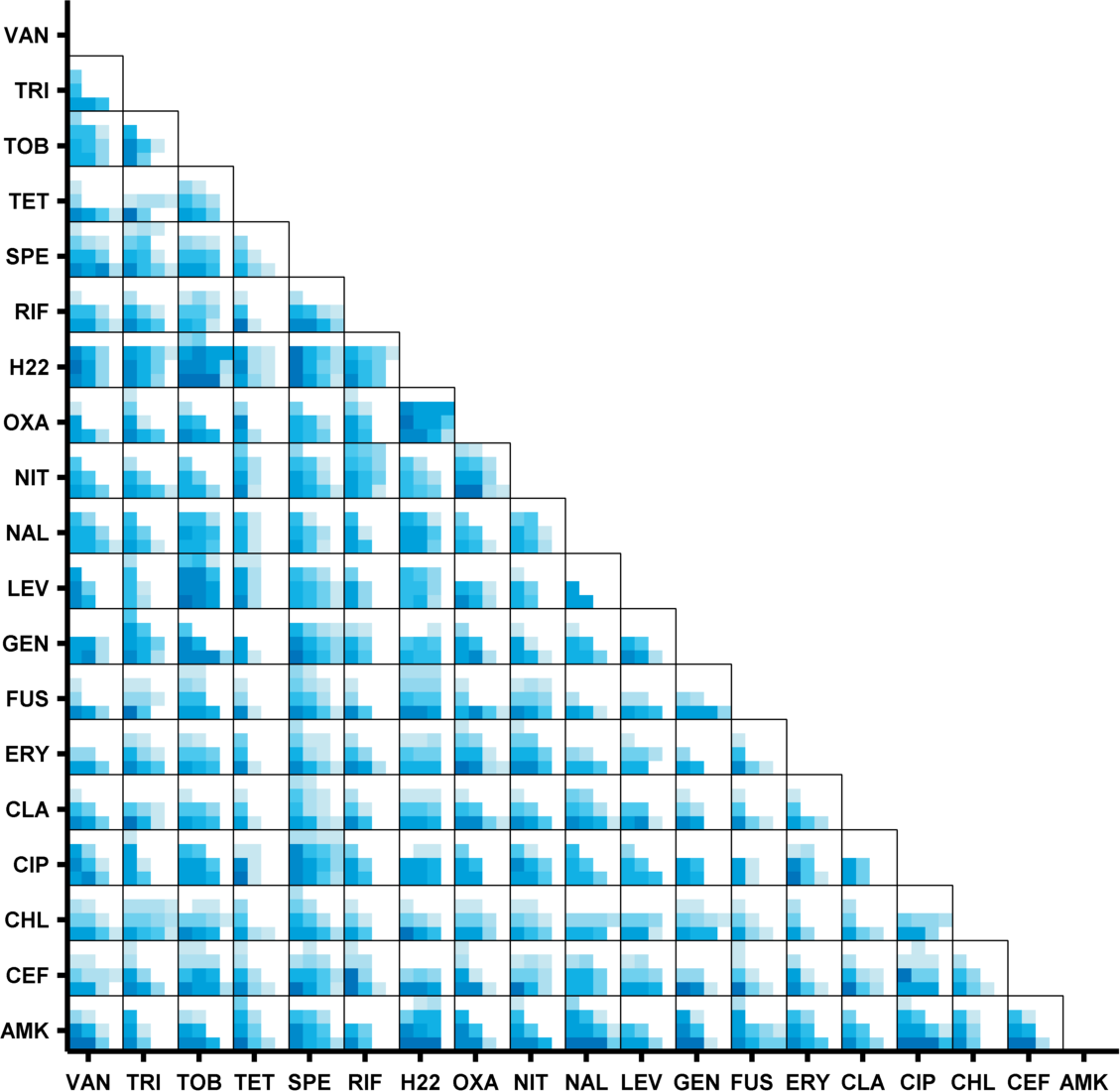

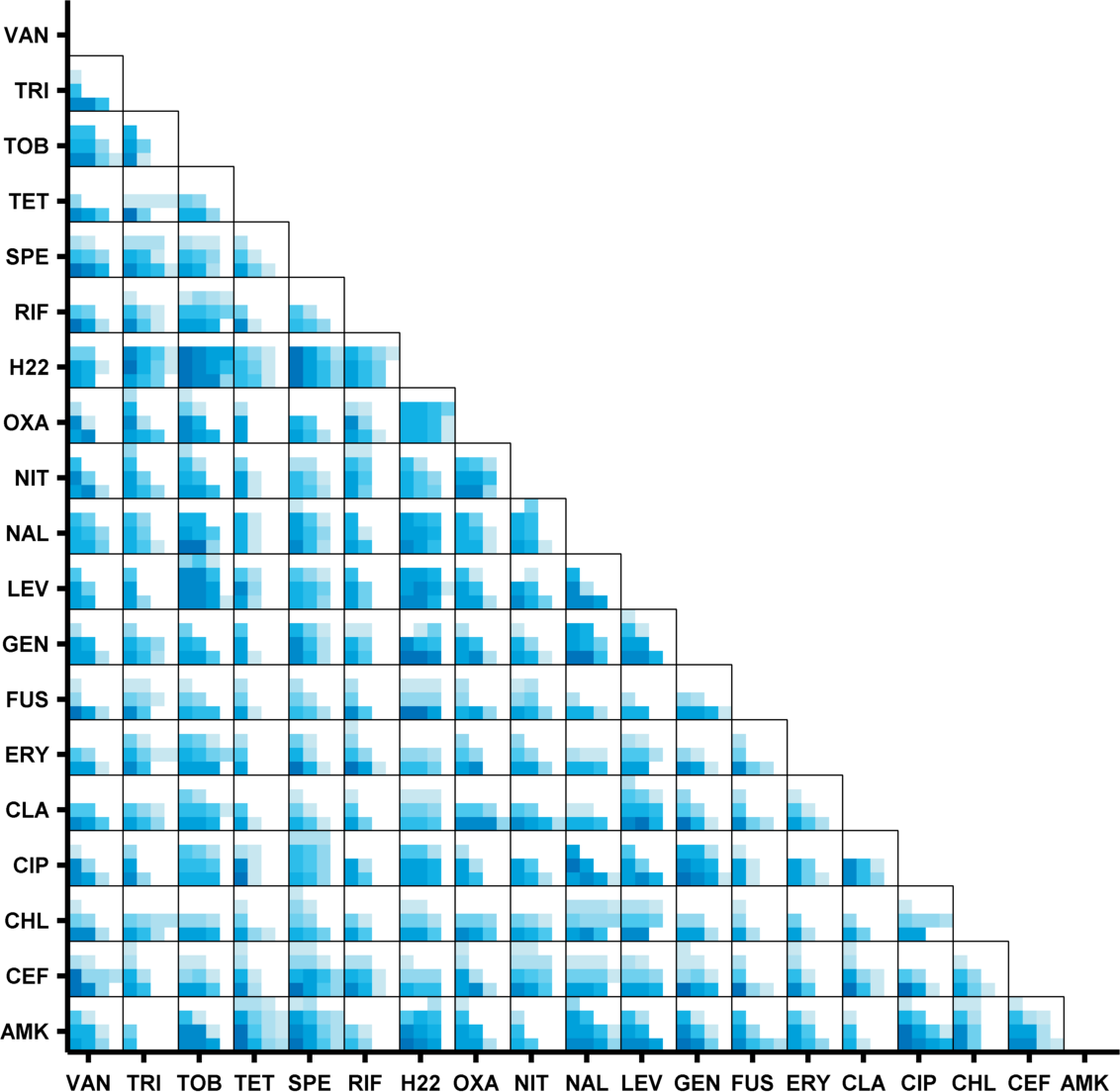
**


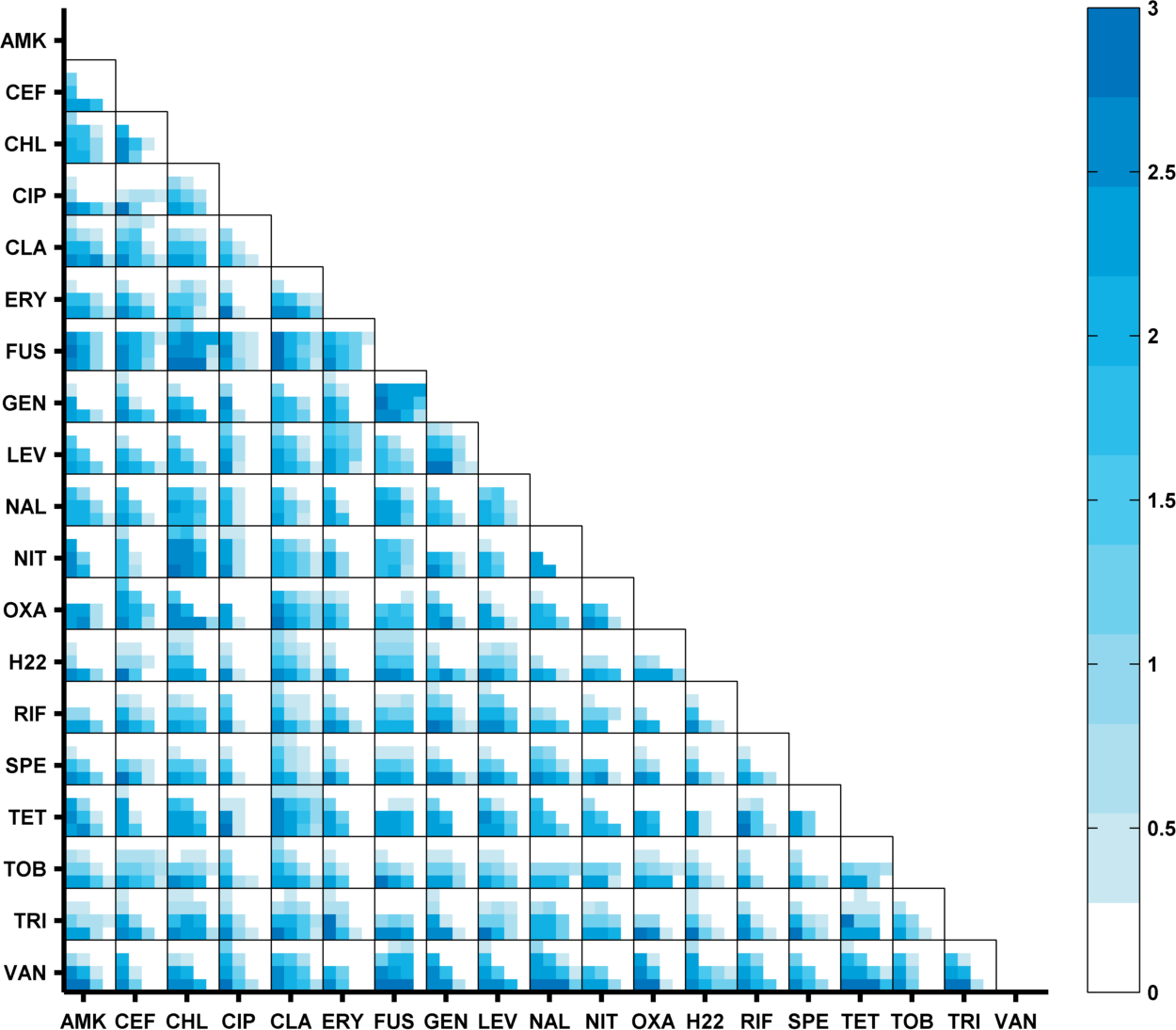


**Replicate 1**

**A**

**Replicate 2**

**
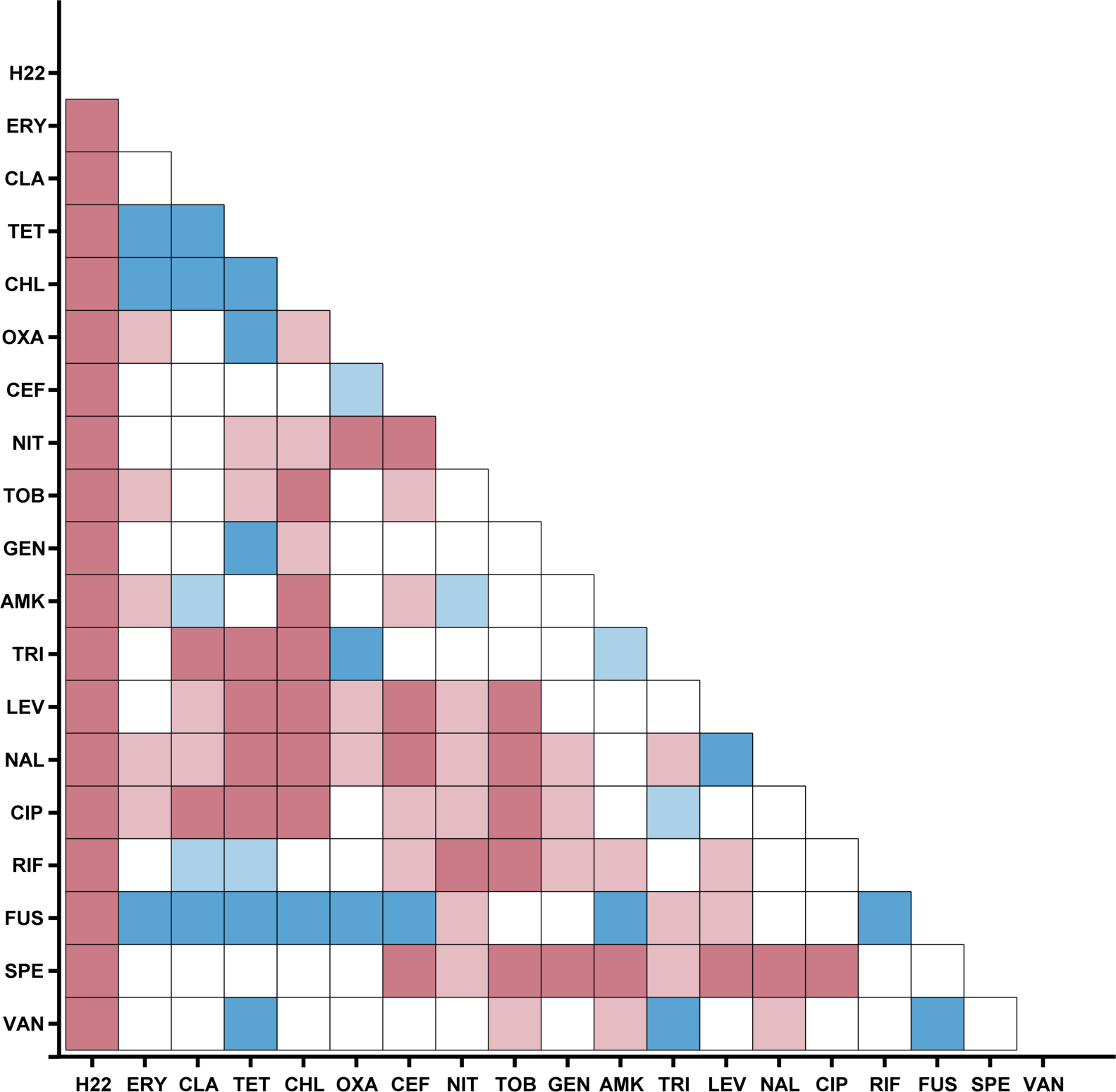
**

**B**

Appendix Figure S1: Growth Data for E. coli grown with different antibiotic combinations (both training and test set). Each drug interaction experiment consisted of 16 growth measurements in various dose combinations of each antibiotic, as described in Methods and Figure 1. For each drug pair, growth rates were measured for all pairwise combinations of four drug concentrations, linearly increasing from 0 to the minimal inhibitory concentration (MIC). The area under the growth curve is plotted for each drug pair at different doses in panel A. Data from both the replicates are shown. This data was used as input to the Loewe’s model to calculate drug interaction scores, shown in panel B.


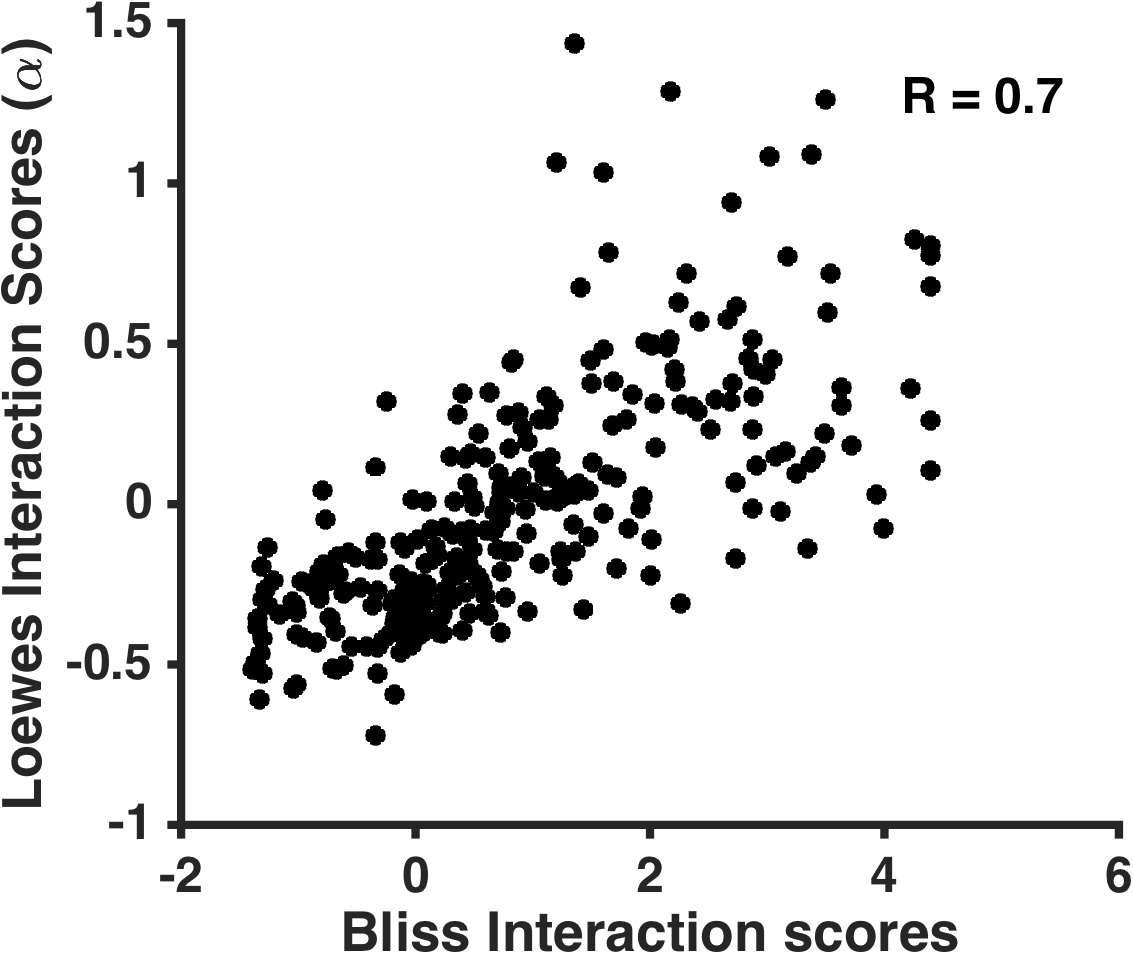


Appendix Figure S2: Concordance between the interaction scores from the Loewe’s Additivity Model and Bliss Interaction Model. Data for all 171 drug combinations are shown. Bliss interaction was quantified for each of the 9 dose combinations for each drug pair and then average value of all dose combination was determined.


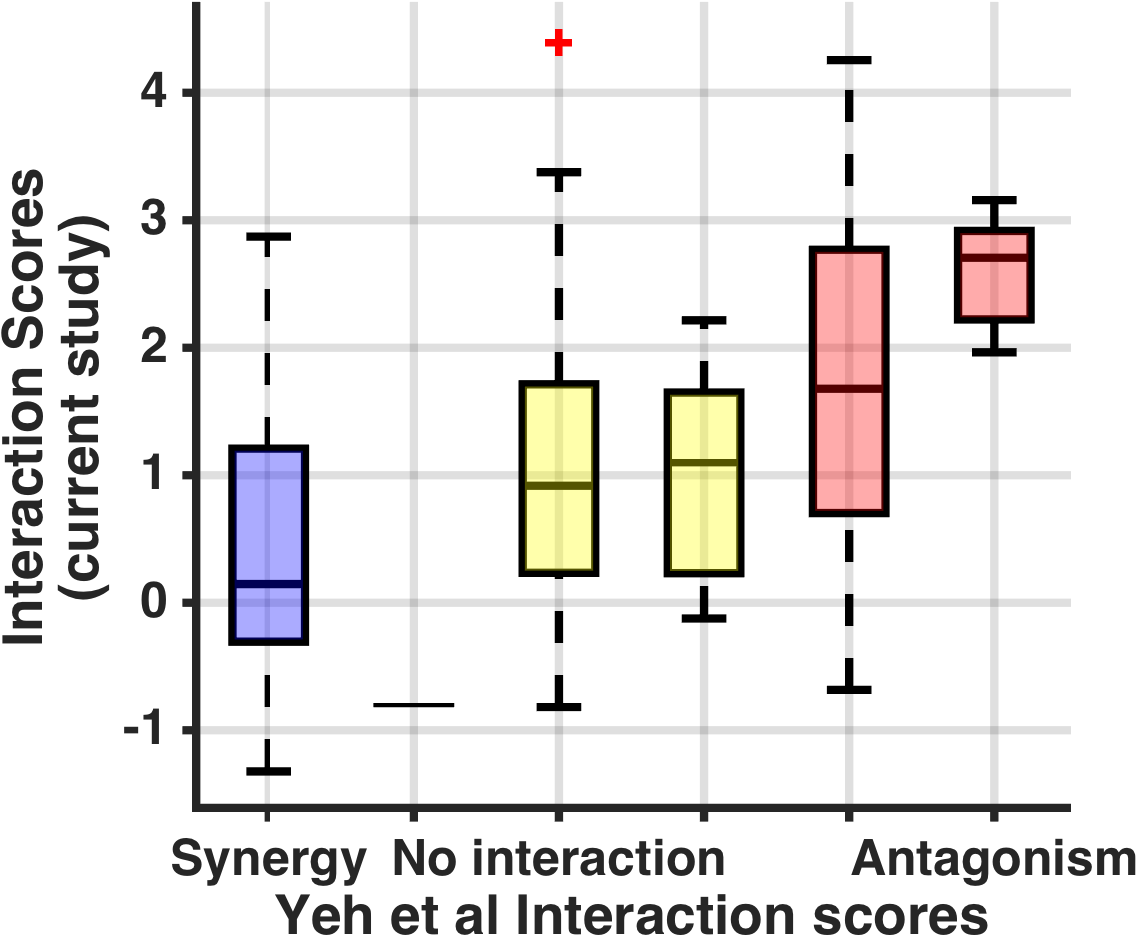

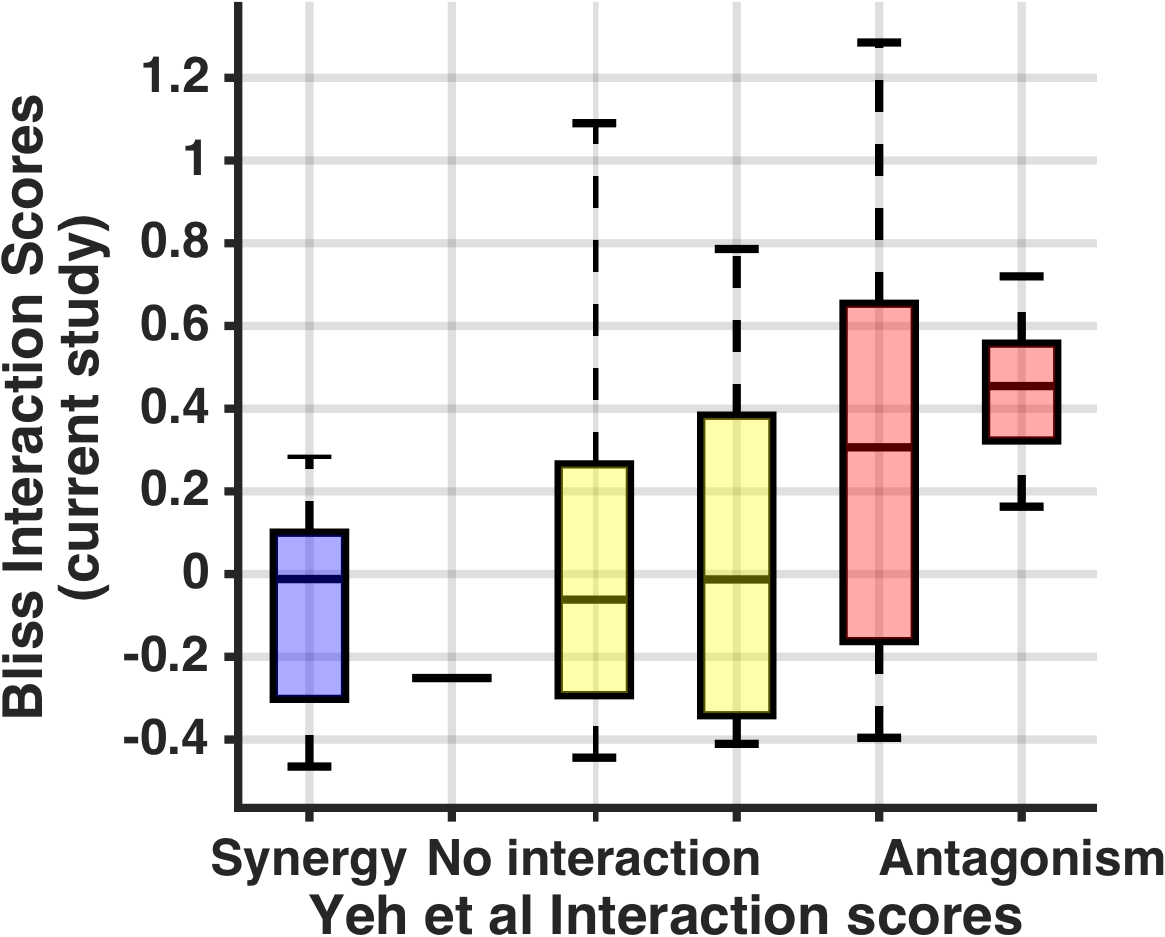


## Appendix Figure S3: Benchmarking experimental data (this study) with Yeh et al drug interaction data set.

We evaluated the drug interaction scores using both the Bliss model (panel B) and the Loewe’s additivity model (panel A), and compared the interaction scores with Yeh et al. We found that the overall correlation between the two data sets among the 66 interactions that were shared was 0.42 (p-value = 0.0004). Among the 66 interactions, only one interaction disagreed completely i.e was synergistic in one data set and antagonistic in the other. 27 were exact matches, while the remaining 38 were predicted to be synergistic or antagonistic in one data set but predicted as non-interacting in the other.

**
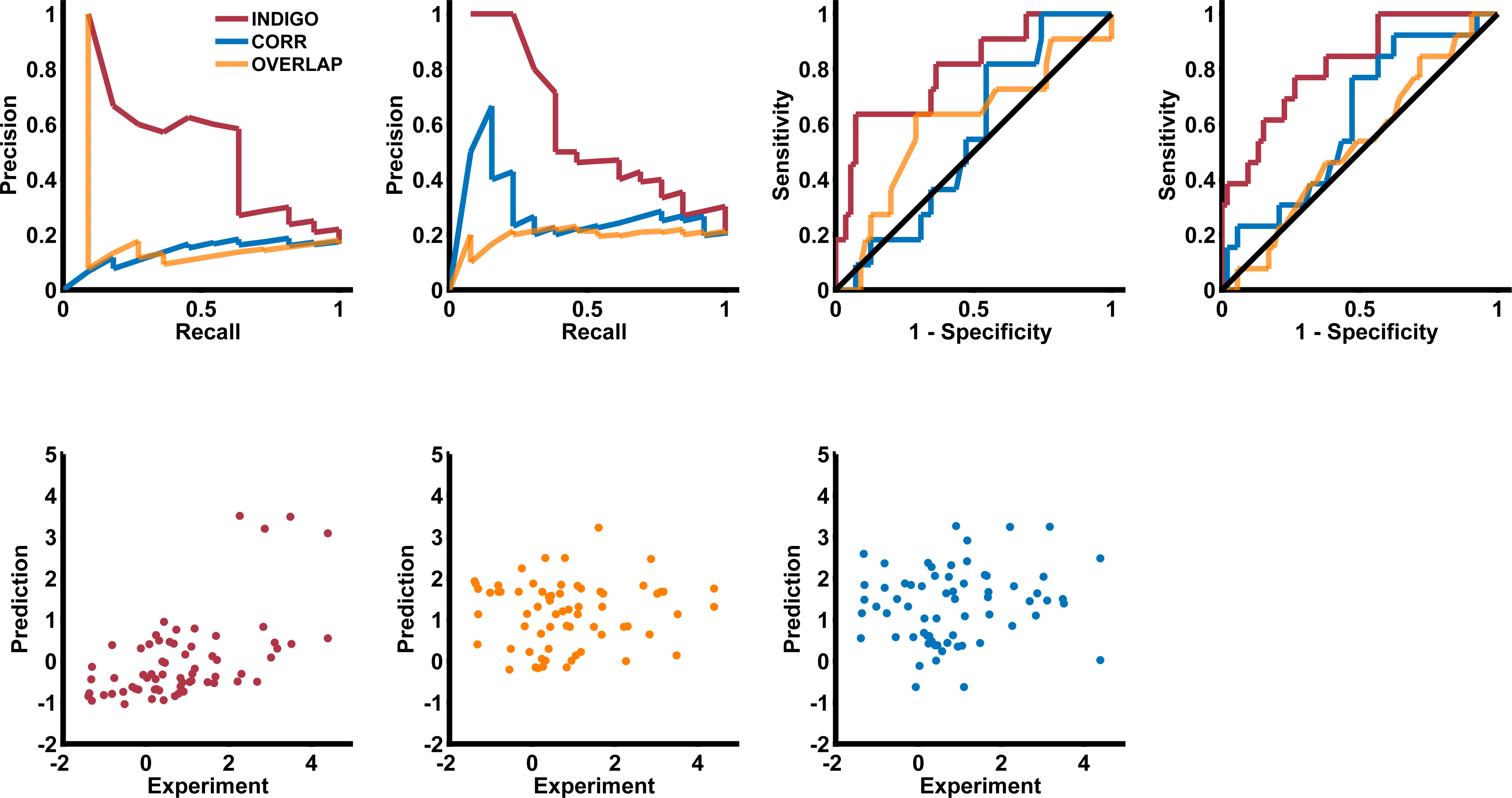
**

**D**

**B**

**C**

**A**

Appendix Figure S4: INDIGO accurately predicts novel drug interactions (ROC curves). INDIGO was evaluated by its ability to predict drug interaction outcomes in in new test data. A. Precision (fraction of identified interactions that are true positives) and Recall (fraction of true positives interactions correctly identified) for INDIGO (shown in red) in predicting synergistic drug interactions. Analogous curves for correlation (blue) and profile overlap (orange) based approaches are shown for comparison. INDIGO achieves at least three times higher precision than other approaches over a range of recall levels. B. Precision - Recall curves for INDIGO (red), correlation (blue) and profile overlap (orange) in predicting antagonistic drug interactions. C and D. Panels show Receiver Operating Characteristic (ROC) curves for INDIGO and profile similarity-based approaches. Plots display sensitivity (true positive rate) and specificity (true negative rate), measured over a range of thresholds for synergy (C) and antagonism (D). All three approaches, INDIGO (red), correlation (blue) and profile overlap (orange), were compared to random prediction (black line; Area Under Curve (AUC) = 0.5). The AUC values for INDIGO were 0.79 (p-value = 10^-16^) for synergy and 0.8 (p-value = 10^-16^) for antagonism

**A**


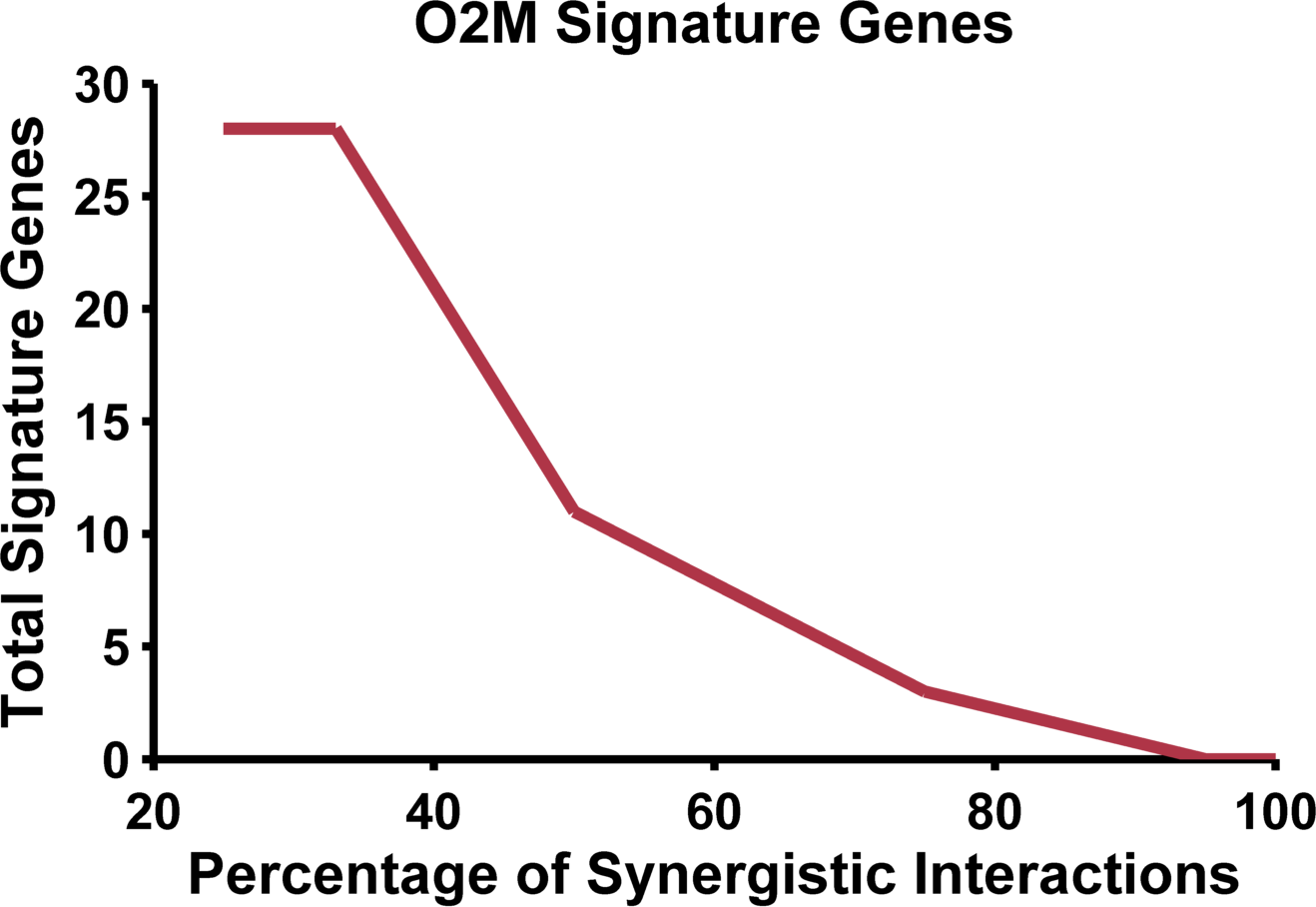


**B**


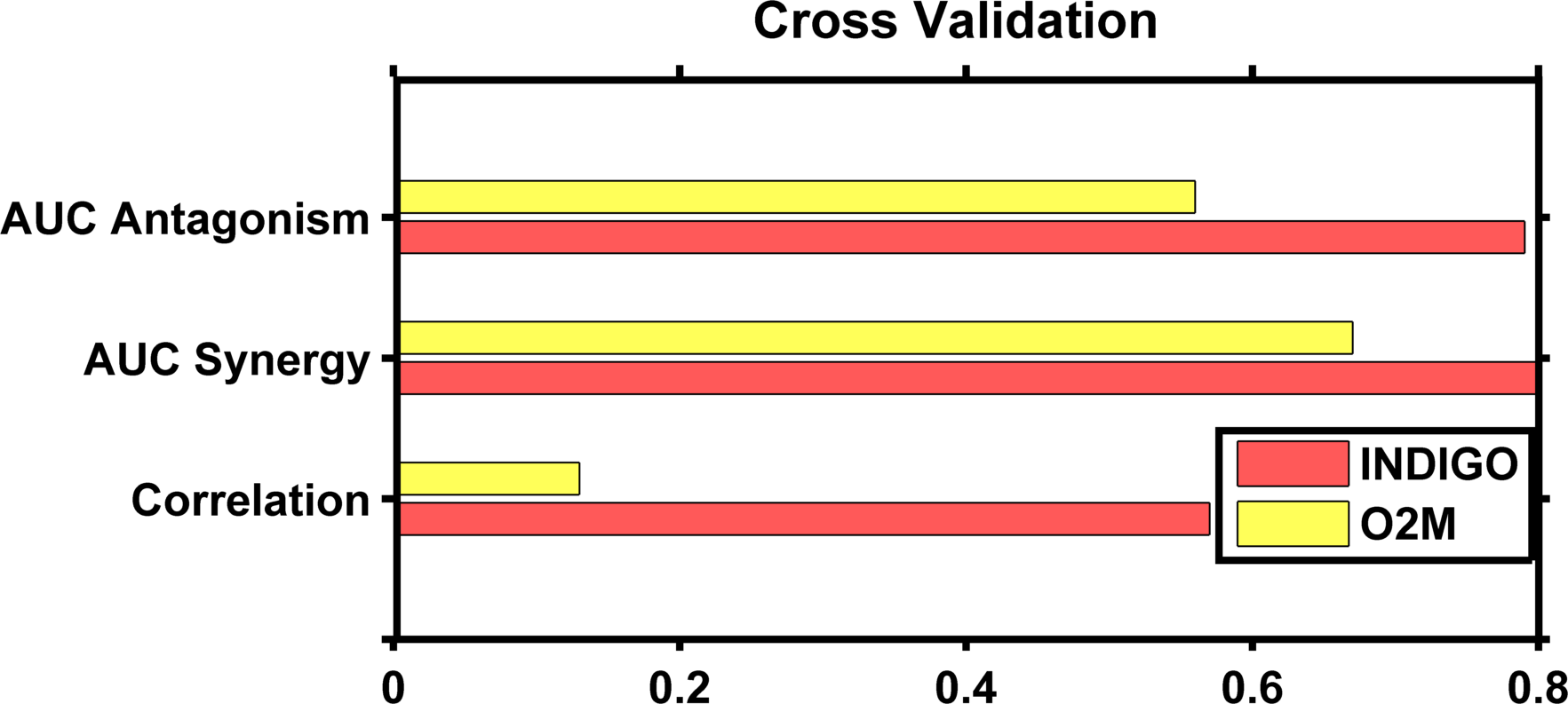


**C**


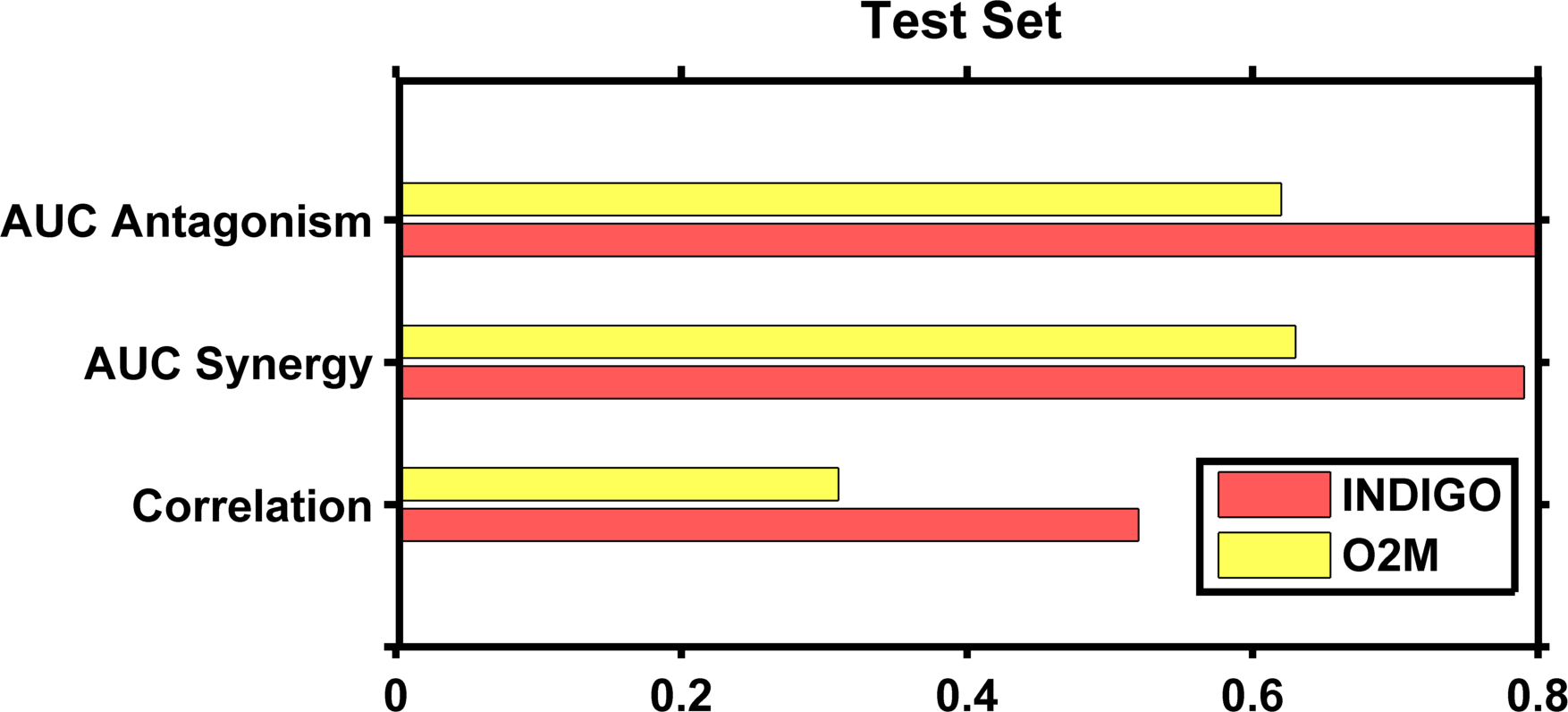


Appendix Figure S5. Comparison with O2M, an overlap-based approach similar to Jansen et al. In O2M a synergy signature is obtained by identifying common genes present in the chemogenomic profiles of synergistic interactions. This synergy signature is then used to predict synergistic interaction outcomes for new drug pairs based on the presence of the synergy signature genes. Given the larger size of our interaction data set (9 strong synergistic interactions in the training set) compared to the training set of O2M (2 synergistic interactions) the original implementation O2M lead to zero signature genes that were present in all synergistic interaction outcomes. The plot in panel A shows total genes that were shared across different fractions of synergistic interactions. Since no genes were present in the chemogenomic profile of all synergistic combinations, we modified the O2M framework and chose genes that were present in at least 75% of the synergistic interactions for prediction in test set. This fraction gave the highest predictive ability based on cross validation in training set and was used for testing in the test set data. The results from this analysis are compared with predictions from INDIGO in leave-one-drug-out cross validation (described in Appendix Figure S9) in the entire data (Panel B) and in test set alone (66 combinations) (Panel C). The low correlation observed for leave-one-drug-out cross validation suggests that similarity and overlap based approaches fail to correctly predict interaction outcomes for new classes of drugs; further they also lack a model for antagonism.


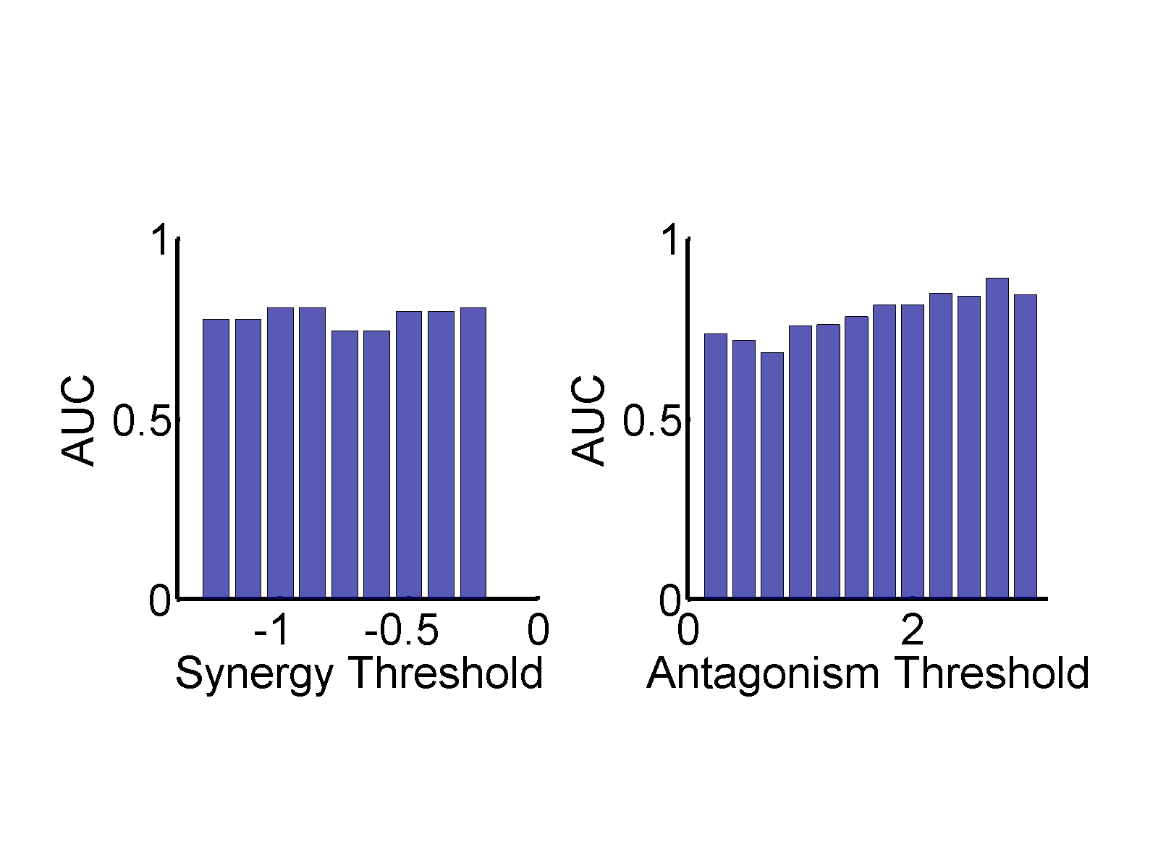


Appendix Figure S6: Effect of changing the threshold for synergy and antagonism. The accuracy of INIDIGO was evaluated by choosing a wide range of thresholds for determining synergy or antagonism. Since INDIGO is trained on quantitative data and makes predictions quantitatively, the choice of the thresholds does not significantly affect performance. The plots demonstrate that changing the thresholds for synergy and antagonism did not significantly affect the AUCs for INDIGO in the test set. This suggests that INDIGO is robust to the choice of parameters chosen for identifying synergy and antagonism.


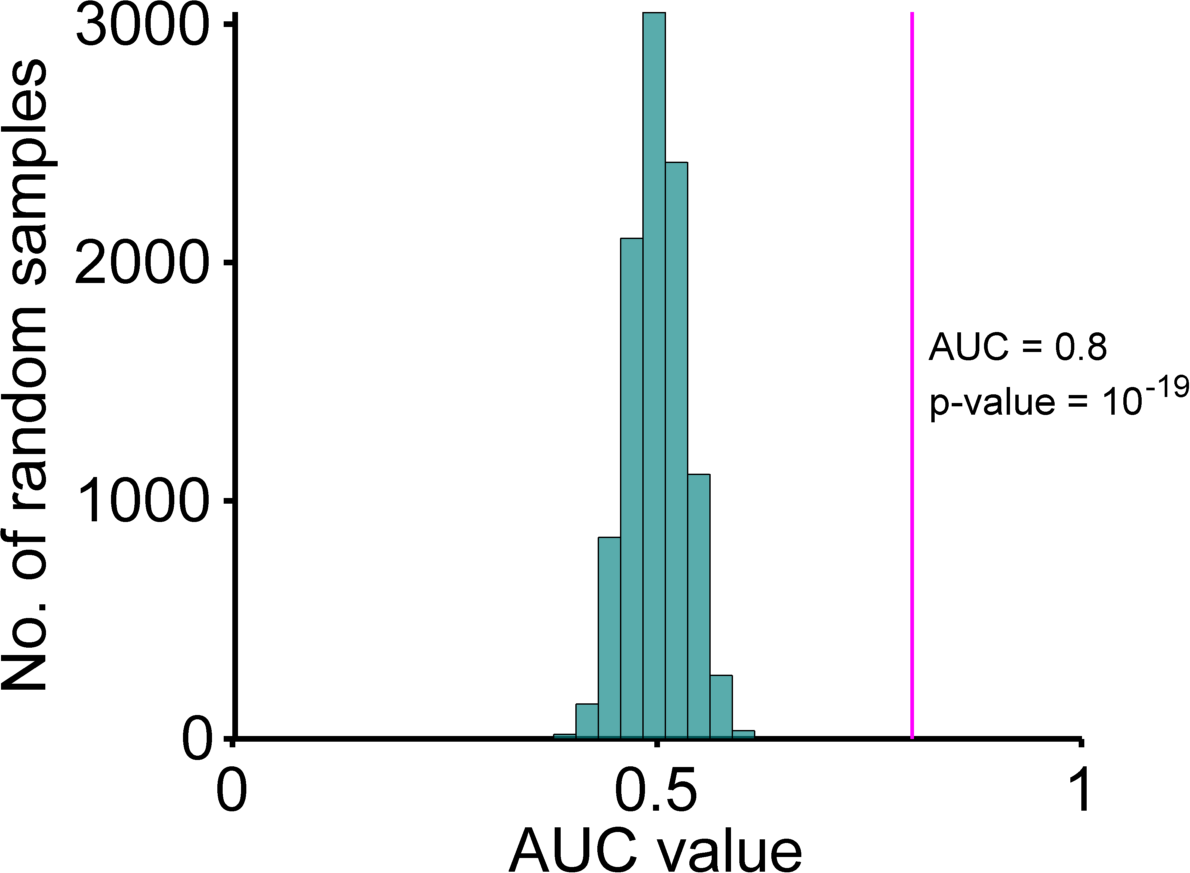


Appendix Figure S7. Estimating statistical significance of predictions by INDIGO by comparing to a random model of drug interaction prediction. The figure shows AUC values for antagonistic predictions obtained by randomly shuffling the alpha scores of experimental interaction data. The predictions from these thousands of random model (shown in green) were compared to actual predictions by INDIGO (shown in pink). A t-test was used to assess the significance of the AUC score obtained by using INDIGO (p-value = 10^-19^). The pink line in the plot represents the AUC for INDIGO in cross validation. P-values for AUC scores for predicting synergy was determined in an analogous fashion. Since the p-values are determined from different distributions for synergy and antagonism, the statistical significance for AUC scores are also correspondingly different for synergy and antagonism.


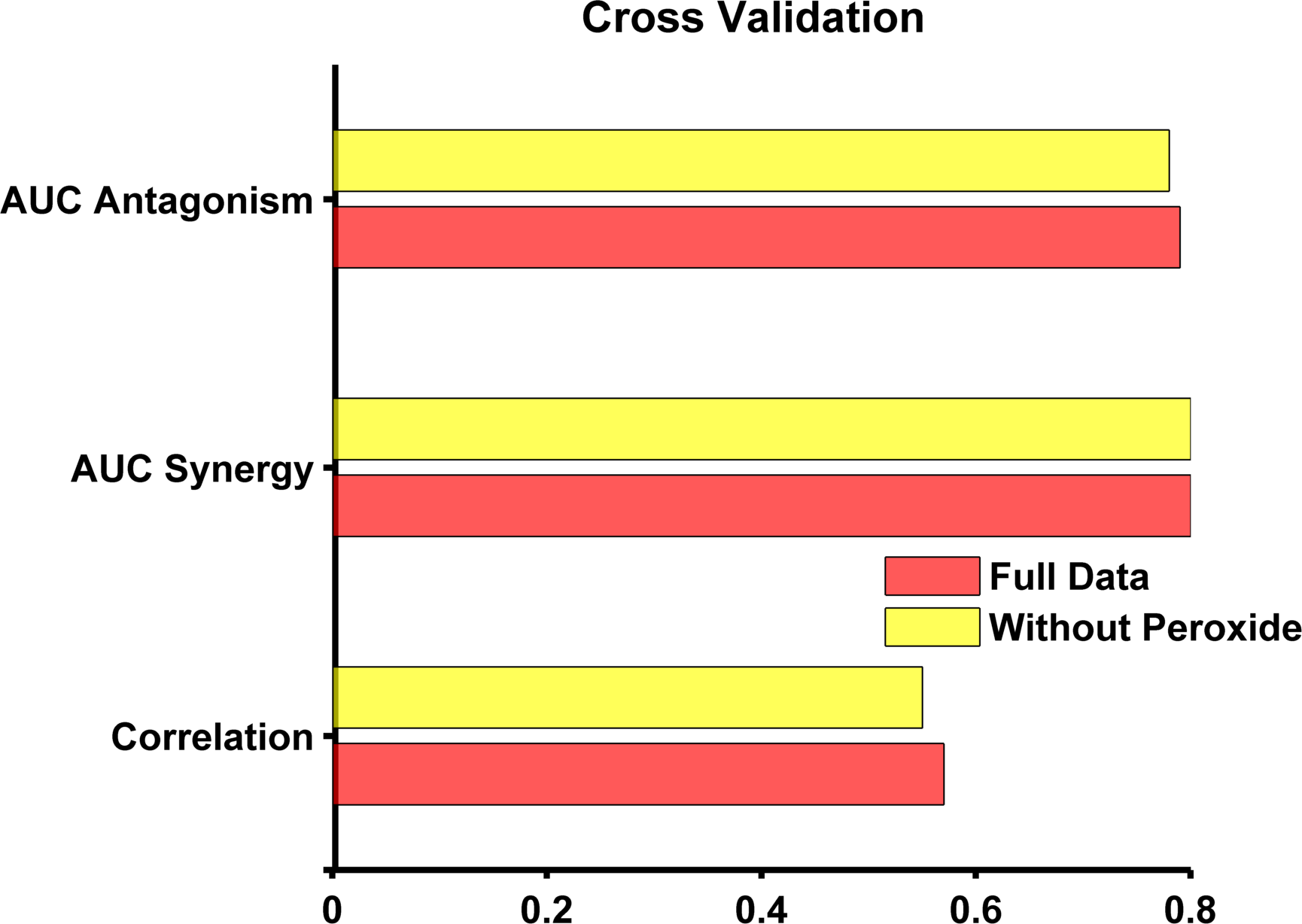


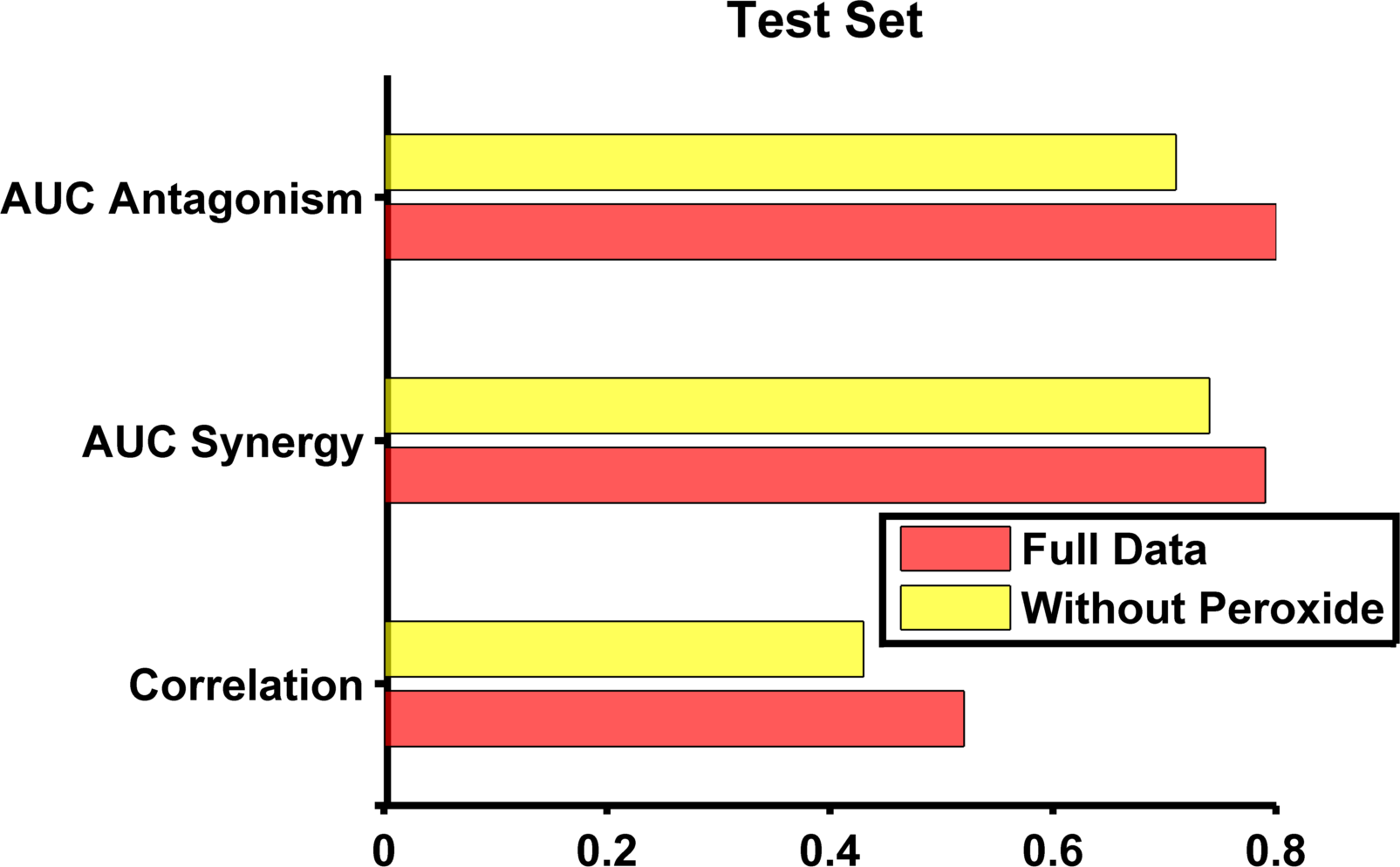


Appendix Figure S8. Effect of removing the outlier hydrogen peroxide on model predictions. Removing hydrogen peroxide from our data set reduced predictive accuracy in test set but not significantly. It did not affect the AUC scores and correlation in leave-one-drug-out cross validation (described in Methods) suggesting that INDIGO is robust to outliers in the data set.


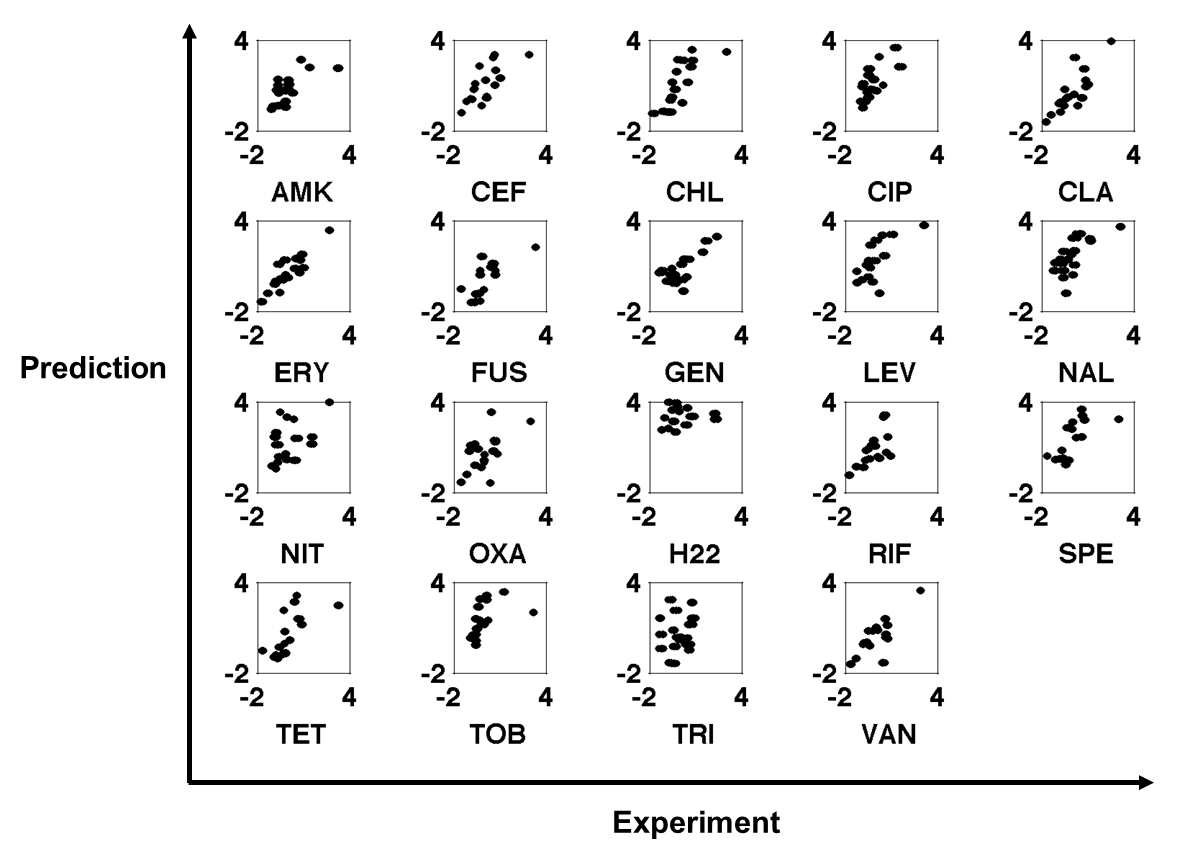


Appendix Figure S9: Cross validation analysis across individual drugs (Leave one drug out cross validation). Each of the 19 drugs in our data set were removed from the drug-interaction network and their interaction was predicted by INDIGO. Interactions of the removed drug was predicted based on its chemogenomic profile and the interaction profile of the remaining drugs in our experimental dataset. This was iteratively repeated for all drugs in our data set and compared against experimental data. This was done to test the robustness of the model, and also to identify drugs that are easier or difficult to predict based on our framework. The average correlation between model prediction and experimental data across all drugs was 0.56. Through this analysis we found that INDIGO can accurately predict interactions with compounds belonging to novel chemical classes or with distinct mechanisms of action. The inability to predict hydrogen peroxide interactions suggest that its mechanism of action is different from what is learned from its chemogenomic profile.


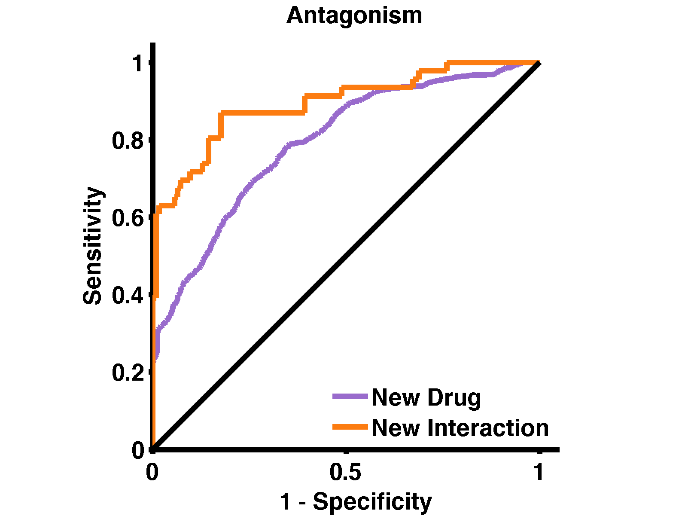

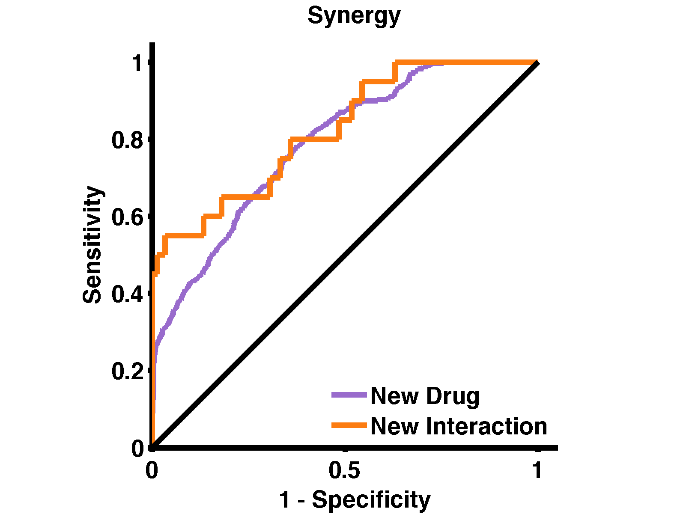


Appendix Figure S10. ROC curves for Ten-fold (orange curve) and Leave-one-drug-out (lavender curve) cross validation. In contrast to the analysis described in Appendix Figure S5, where a drug of interest was removed from the drug interaction network and its interactions inferred by INDIGO (lavender curve), we found that the presence of a few known interactions about a drug of interest can greatly improve the prediction accuracy (orange curve). Results are shown based on ten-fold cross validation, where 10% of the data is blinded and used as a test set to evaluate the accuracy of the model. Our results suggests that including a few interactions for each drug in the training data improves test set prediction accuracy.


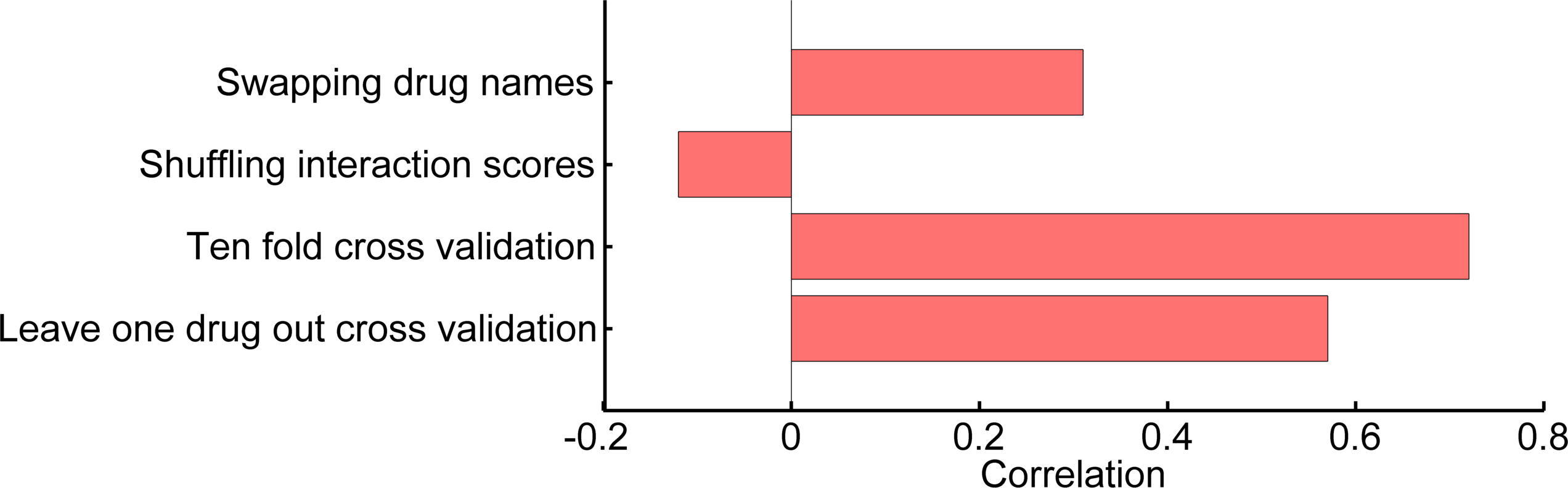


Appendix Figure S11. Controls and cross validation analyses to test INDIGO**.** Average test set correlation obtained via ten-fold and leave one drug out cross validation analysis (described in methods) are shown. Label descriptions: **Swapping drug names** – In this analysis the identity of the drug names in the chemogenomic data were swapped during input to INDIGO, which resulted in switching the chemogenomic profiles of individual drugs. This analysis led to a significant decrease in the test set correlation, confirming that chemogenomic profiles provide unique drug-specific information. **Shuffling interaction scores** - We randomly permuted the interaction scores used for training INDIGO and found that, as expected, it greatly reduced the predictive ability of the model in test set. **Ten fold cross validation** & **Leave one drug out cross validation** - As described in the methods section, a small fraction of the interaction data is hidden from INDIGO and the model predicts the interaction outcomes for the hidden fraction of the data, based on the available (non-hidden) data set.


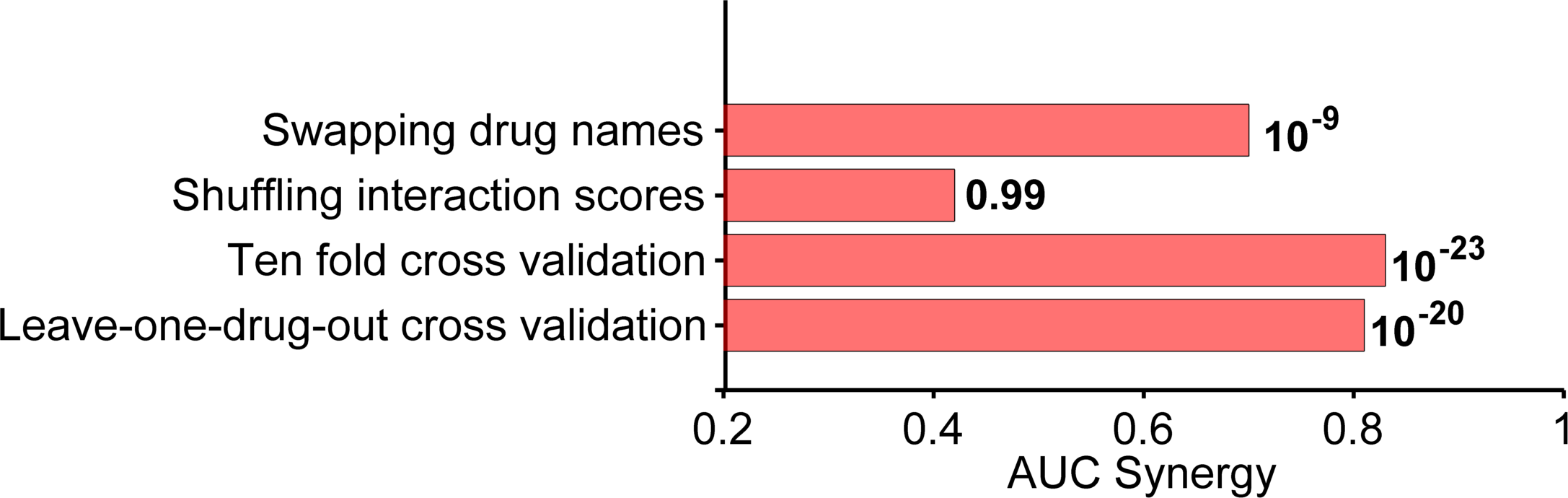


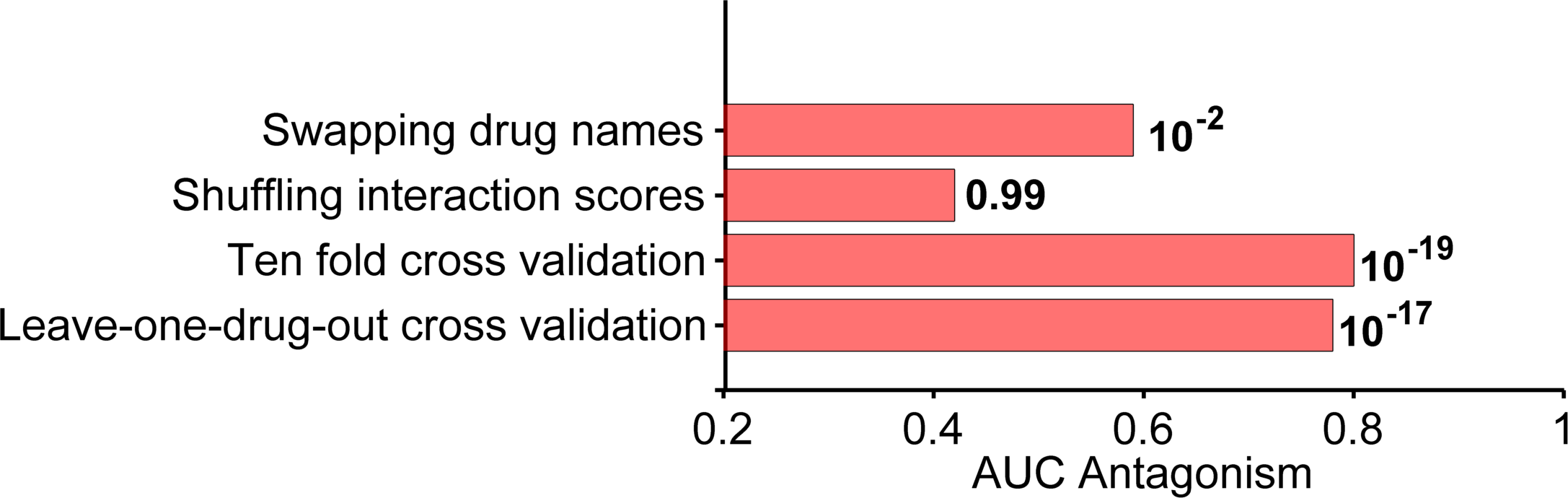


Appendix Figure S12. Controls and cross validation analyses to test INDIGO (AUC values). AUC values for predicting synergistic and antagonistic interactions, and the corresponding p-values based on comparison with a random model are shown. The row label categories are described in Appendix Figure S10.


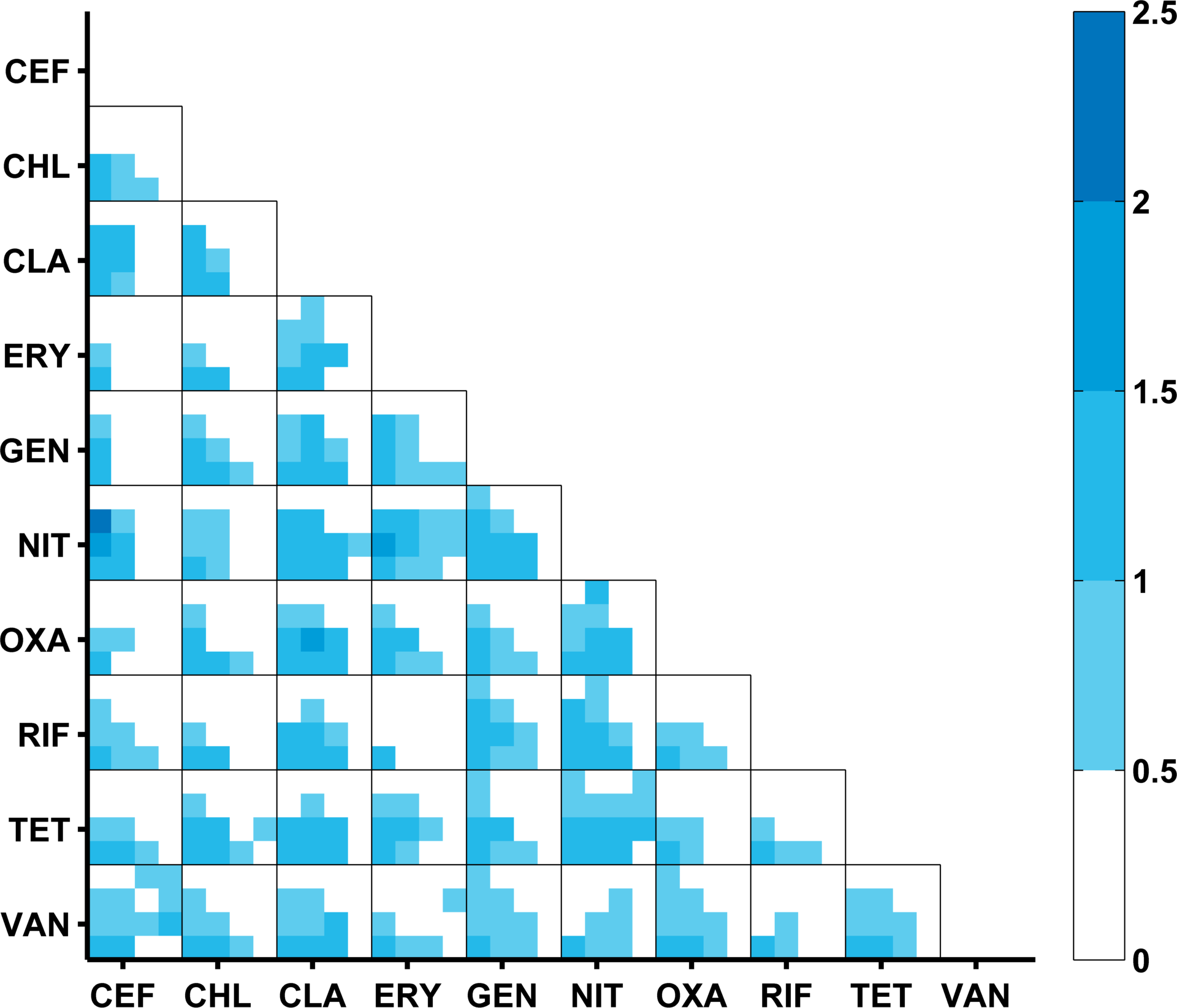

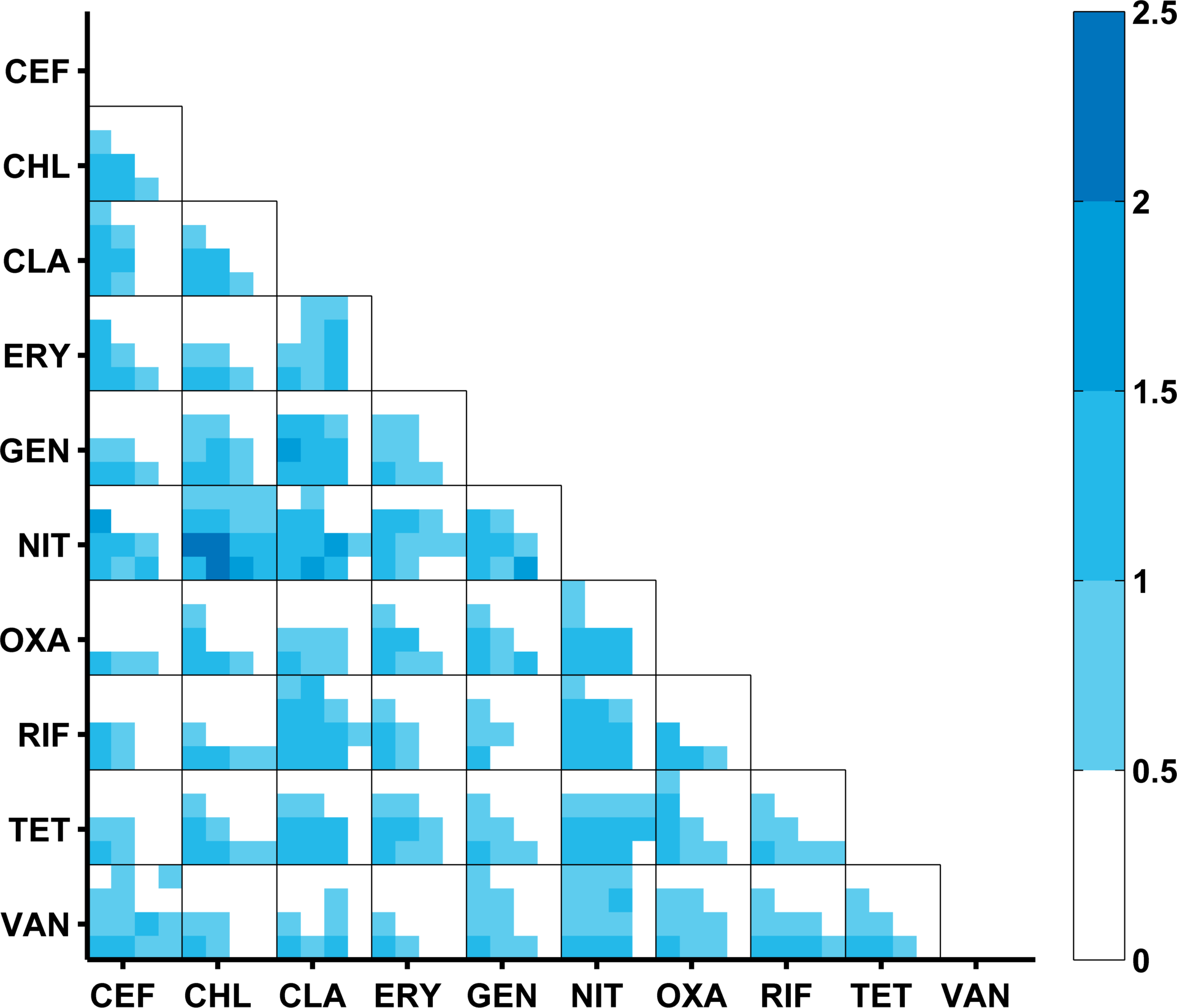


**
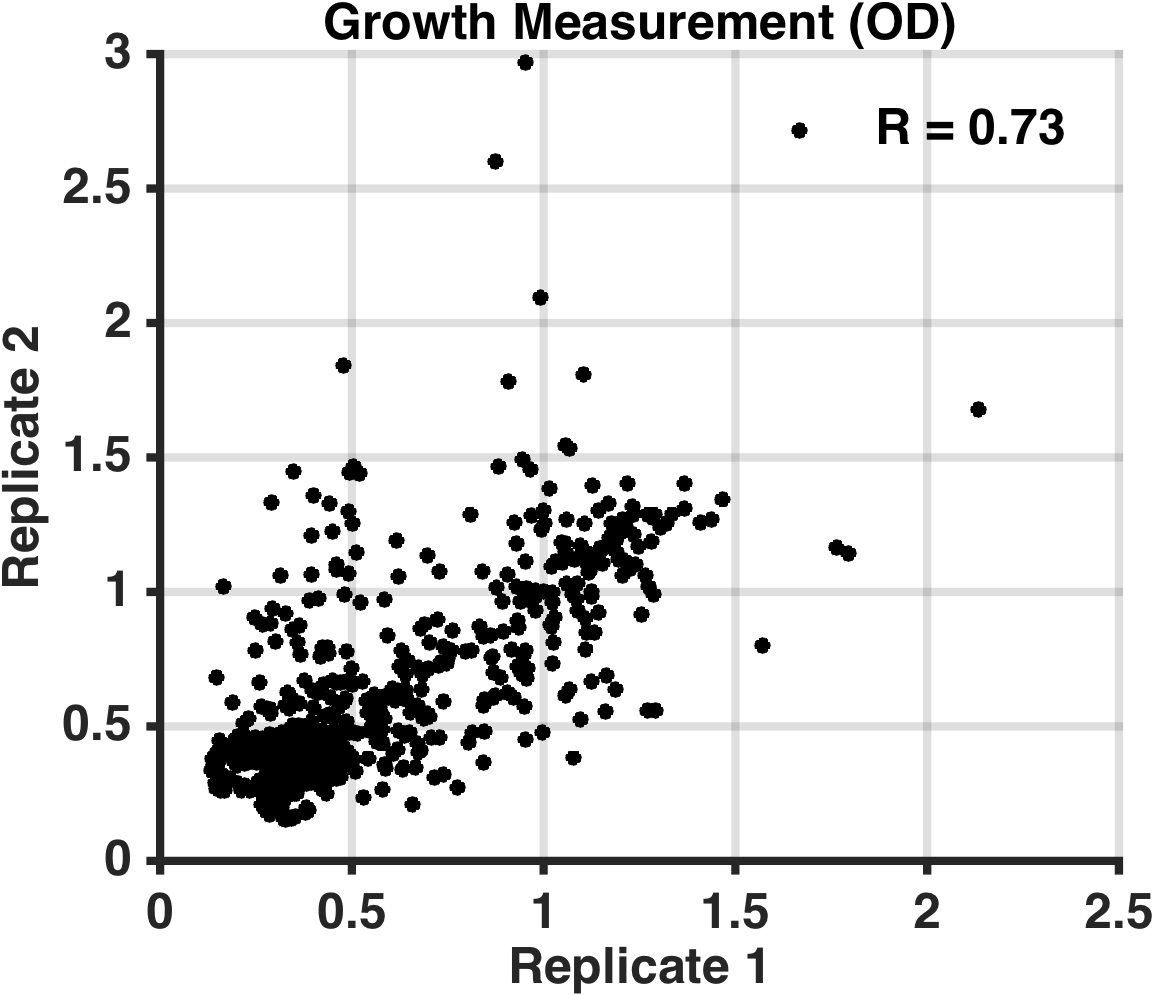

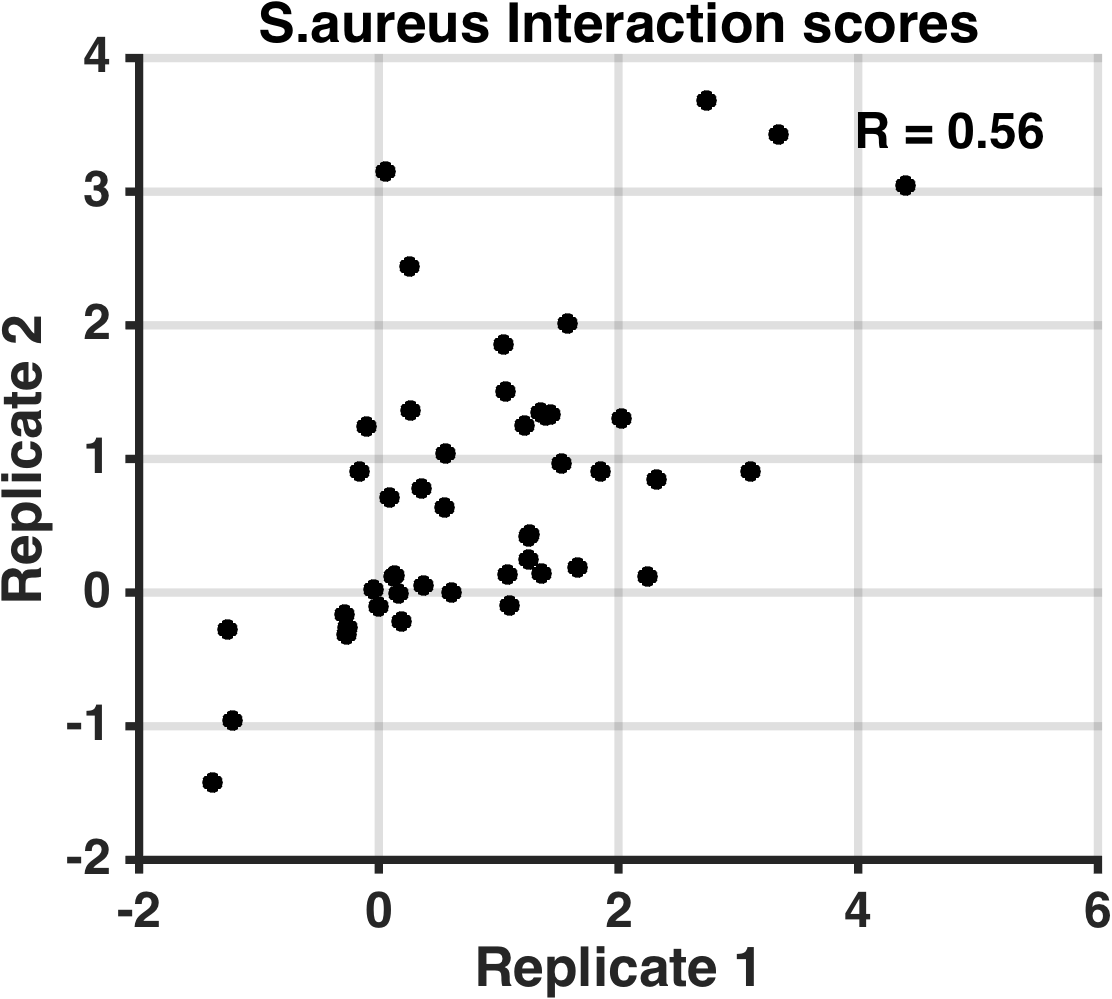
**

D

C

Appendix Figure S13: Growth Data for S. aureus grown with different antibiotic combinations. *S. aureus* cells were grown in the presence of different pairs of antibiotics. Each pairwise interaction experiment consisted of 16 dose combinations of individual drugs, as described in Methods and Figure 1. For each drug pair, growth rates were measured for all pairwise combinations of four drug concentrations, linearly increasing from 0 to the minimal inhibitory concentration (MIC). The final OD measurement after 12 hours is plotted for each drug pair at different doses. Data from two replicates are shown. This data was used as input to the Loewe’s model to calculate drug interaction scores. The correlation between AUC growth values between replicates was 0.73 (panel C) and the correlation between alpha scores was 0.56 (p-value = 10^-5^) (panel D).


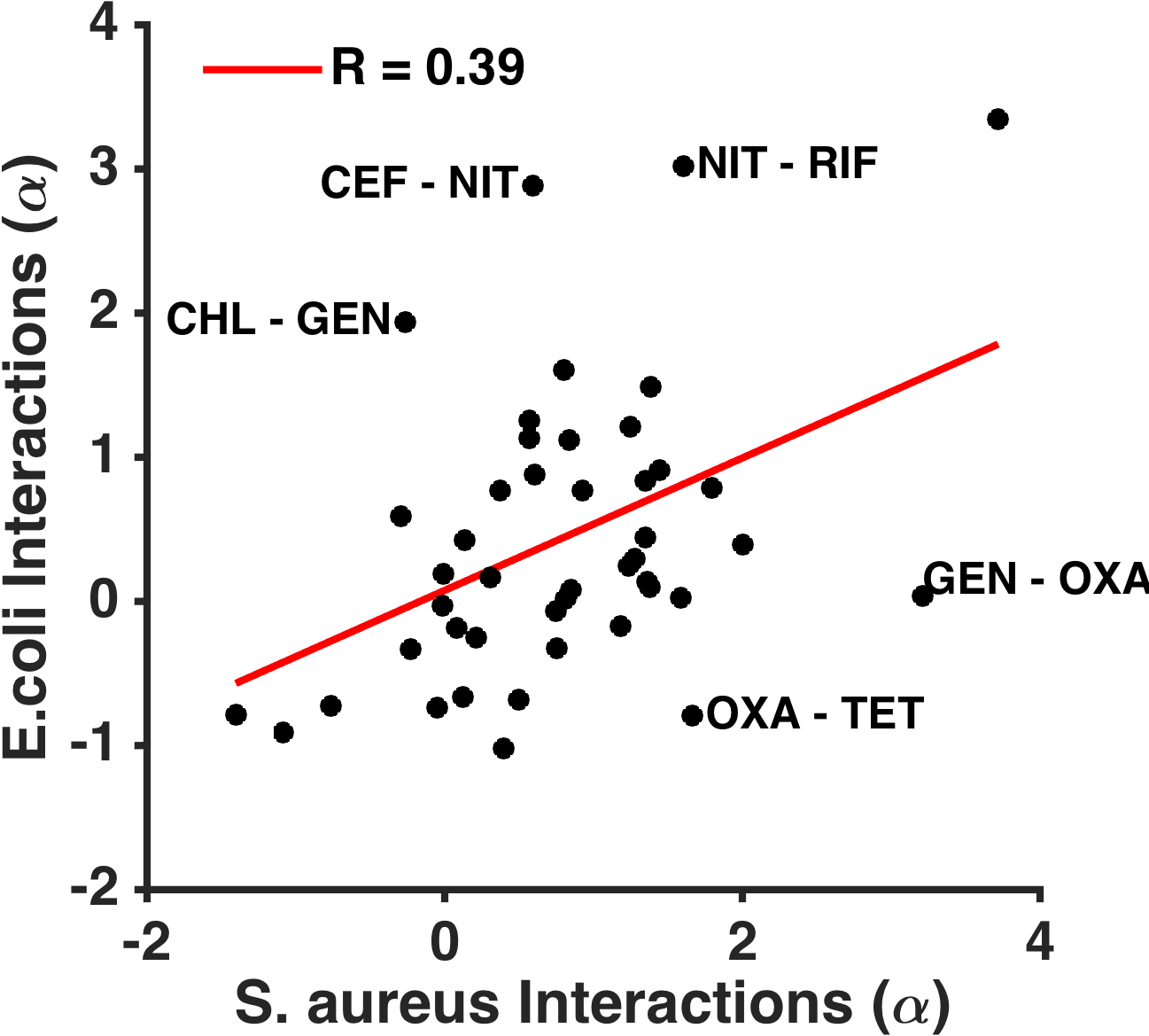

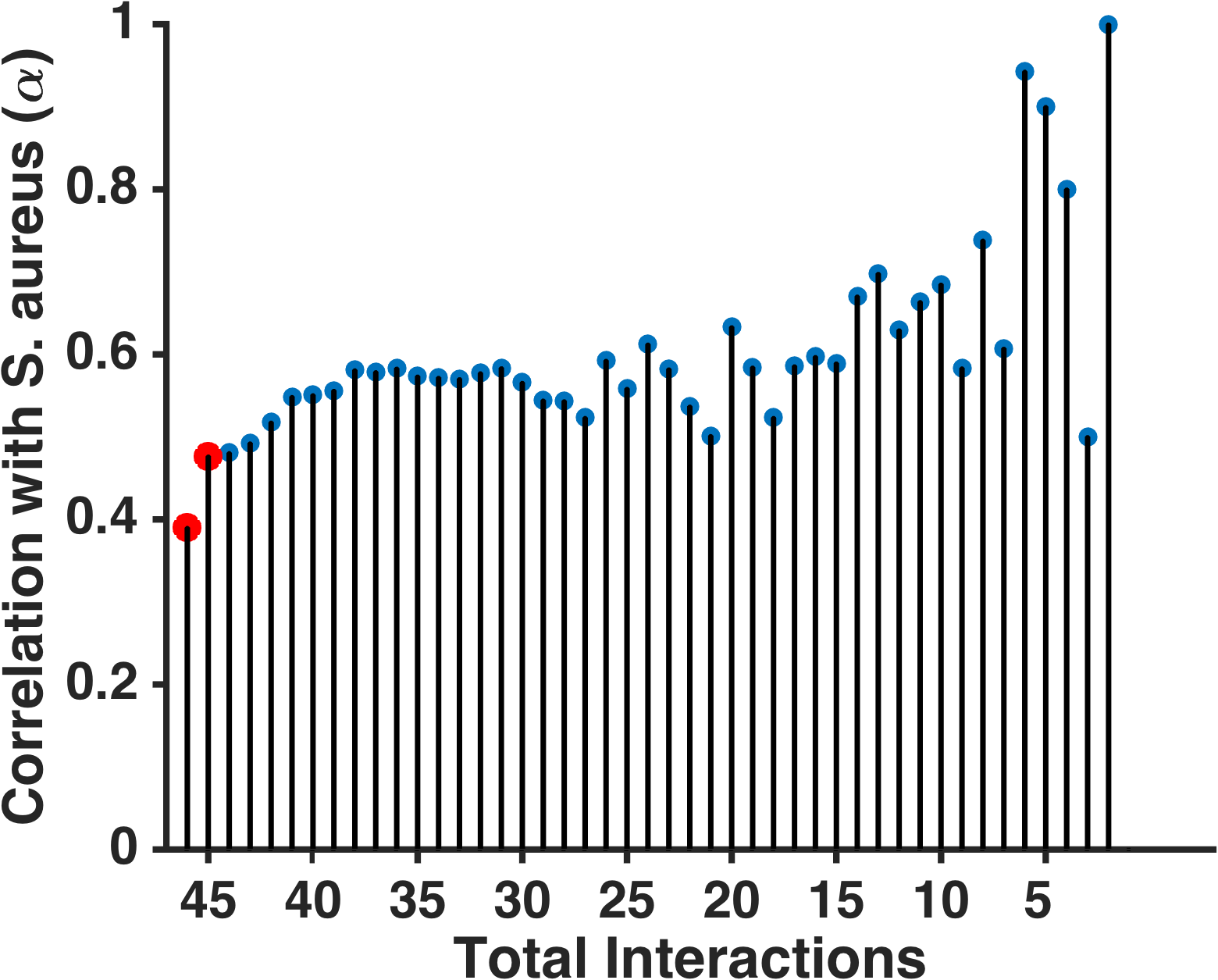


Removing variable interactions increases correlation

A

B

Appendix Figure S14: A. Scatter plot of experimentally measured interaction scores of the two species. Outliers and strong synergistic and antagonistic interactions are highlighted. B. Identifying subset of conserved interactions between species using INDIGO**.** By prioritizing interactions based on the magnitude of predicted difference by INDIGO, we can identify conserved interactions and better predict interaction outcomes in *S. aureus.* The red data points are the predictions using entire *E. coli* data (R = 0.39; p-value = 0.008) and using *E. coli* data + INDIGO predictions (R = 0.47).


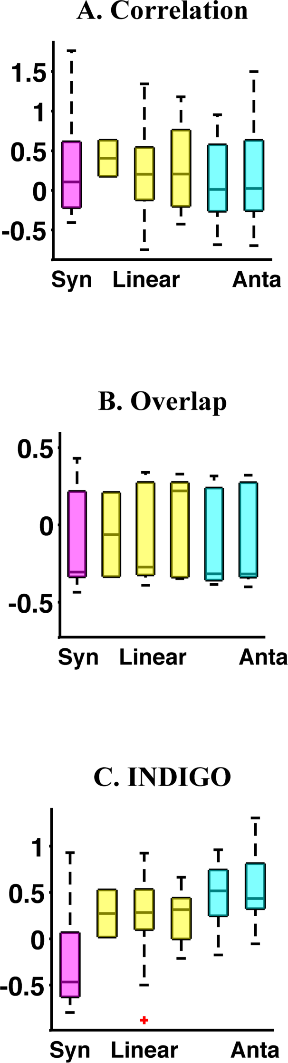

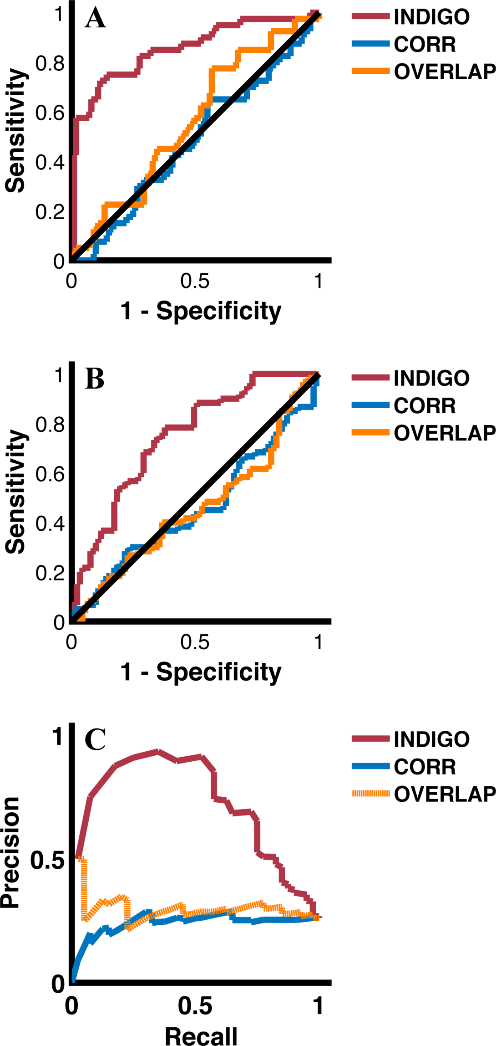


**D**

**E**

**F**

Appendix Figure S15. INDIGO accurately predicts drug interactions in an independent study. INDIGO was evaluated by its ability to predict drug interaction outcomes using data from Yeh et al (Yeh et al, 2006), involving 21 antibiotics targeting diverse cellular processes. Of the 21 antibiotics, 18 had chemogenomic data in Nichols et al. Boxplots A, B and C show measured interactions and predictions by INDIGO (panel C), profile overlap (panel B) and correlation (panel A). Interactions are ordered based on most synergistic (score = -1) to the most antagonistic (score = 1) or suppressive (score = 2). INDIGO quantitatively predicted drug interactions with high accuracy in test conditions based on cross validation (correlation - 0.59, p-value < 10^-15^; AUC – 0.85 & 0.75, p-value – 10^-12^ & 10^-7^ for synergy and antagonism respectively). Both the correlation and profile overlap based approaches were significantly less accurate than INDIGO in predicting drug interactions (Correlation = 0.06 & 0.07, AUC for synergy – 0.56 & 0.57, p-value = 0.23 & 0.16; for correlation and profile overlap respectively). D. Receiver Operating Characteristic (ROC) curves for INDIGO and profile similarity-based approaches. Plots show sensitivity (true positive rate) and specificity (true negative rate), measured over a range of thresholds for synergy. The corresponding curves for antagonism are shown in panel E. F. Precision and Recall for INDIGO (shown in red) in predicting synergistic drug interactions. Analogous curves for correlation (blue) and profile overlap (orange) based approaches are shown for comparison.

**Interactions between the same drug at different doses**


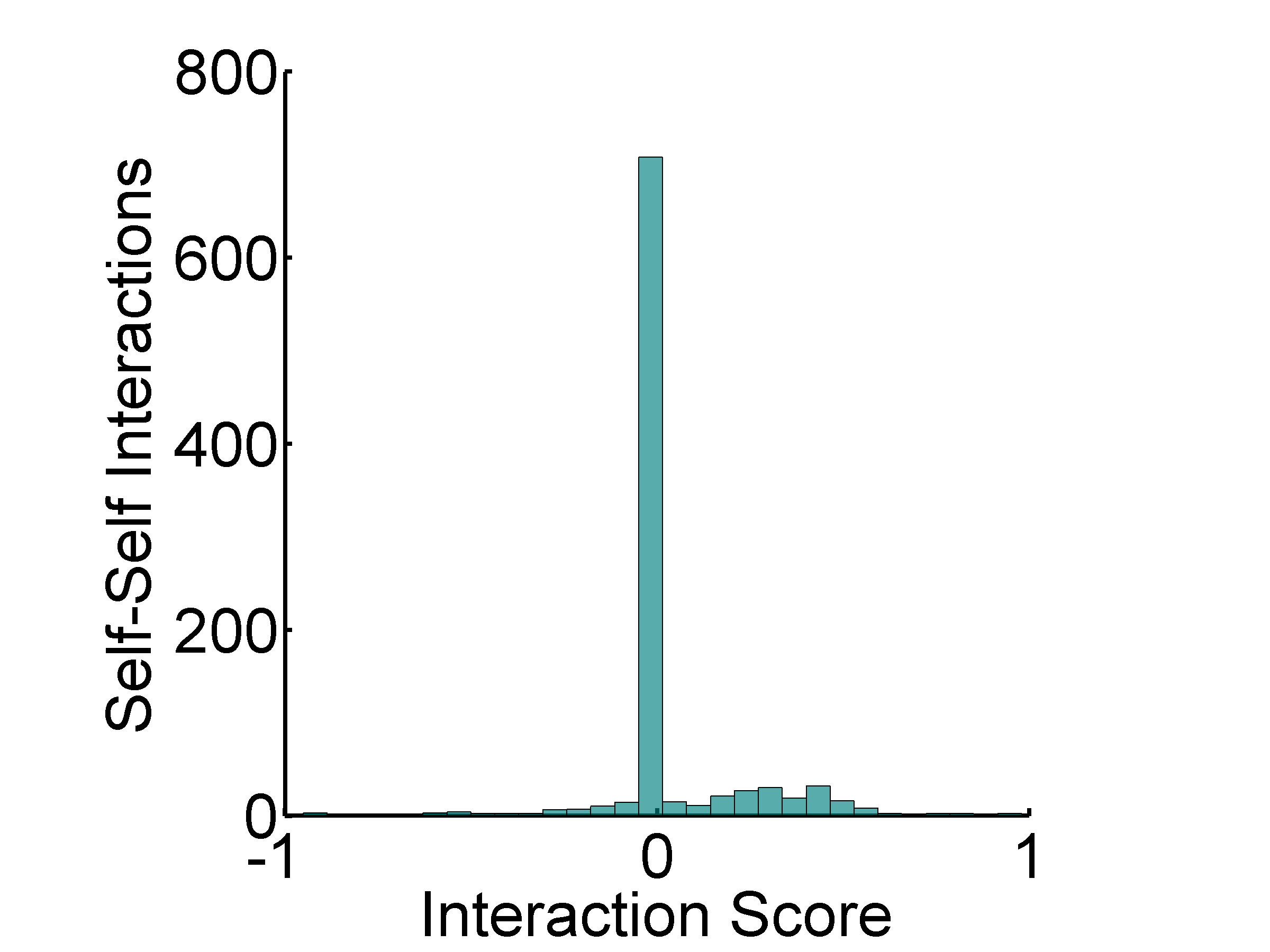


Total no. of Interactions

Appendix Figure S16: Positive control for running INDIGO**.** INDIGO correctly predicted interactions between the same drug at different doses to be linear (median score = 0). Chemogenomic profiles for 73 drugs from Nichols et al at different doses were used to determine interactions between the same drug at different doses. Data for 951 interactions between the same drugs at different doses is displayed in the histogram. Despite the bias in the training data with more antagonistic interactions than synergistic interactions, INDIGO accurately predicted self-self interactions of the same drug at different doses (represented by different chemogenomic profiles) to be non-interacting.

# Appendix Tables:

| Drug 1 | Drug 2 | Predictions | Experiment |
| --- | --- | --- | --- |
| FUSIDICACID-50 | PEROXIDE-2.0 | 3.51 | 2.27 |
| PEROXIDE-2.0 | VANCOMYCIN-50 | 3.49 | 3.49 |
| PEROXIDE-2.0 | SPECTINOMYCIN-4.0 | 3.20 | 2.87 |
| PEROXIDE-2.0 | RIFAMPICIN-1.0 | 3.09 | 4.39 |
| NALIDIXICACID-2.0 | RIFAMPICIN-1.0 | 0.95 | 0.44 |
| NALIDIXICACID-2.0 | SPECTINOMYCIN-4.0 | 0.83 | 2.84 |
| NALIDIXICACID-2.0 | VANCOMYCIN-50 | 0.79 | 1.18 |
| FUSIDICACID-50 | NALIDIXICACID-2.0 | 0.76 | 0.73 |
| CHLORAMPHENICOL-1.5 | RIFAMPICIN-1.0 | 0.63 | 0.24 |
| LEVOFLOXACIN-0.002 | RIFAMPICIN-1.0 | 0.61 | 1.69 |
| SPECTINOMYCIN-4.0 | TOBRAMYCIN-0.4 | 0.55 | 4.39 |
| LEVOFLOXACIN-0.002 | VANCOMYCIN-50 | 0.50 | 0.32 |
| CIPROFLOXACIN-0.004 | VANCOMYCIN-50 | 0.46 | 0.57 |
| LEVOFLOXACIN-0.002 | SPECTINOMYCIN-4.0 | 0.45 | 3.11 |
| CIPROFLOXACIN-0.004 | RIFAMPICIN-1.0 | 0.42 | 0.67 |
| CIPROFLOXACIN-0.004 | SPECTINOMYCIN-4.0 | 0.42 | 3.51 |
| CHLORAMPHENICOL-1.5 | VANCOMYCIN-50 | 0.41 | 0.08 |
| CHLORAMPHENICOL-1.5 | FUSIDICACID-50 | 0.39 | -0.82 |
| FUSIDICACID-50 | LEVOFLOXACIN-0.002 | 0.35 | 1.10 |
| CHLORAMPHENICOL-1.5 | SPECTINOMYCIN-4.0 | 0.31 | -0.12 |
| RIFAMPICIN-1.0 | TOBRAMYCIN-0.4 | 0.30 | 3.17 |
| CIPROFLOXACIN-0.004 | FUSIDICACID-50 | 0.16 | 0.95 |
| TOBRAMYCIN-0.4 | VANCOMYCIN-50 | 0.12 | 1.60 |
| NITROFURANTOIN-2.0 | RIFAMPICIN-1.0 | 0.09 | 3.02 |
| NITROFURANTOIN-2.0 | SPECTINOMYCIN-4.0 | 0.03 | 1.72 |
| NITROFURANTOIN-2.0 | VANCOMYCIN-50 | -0.01 | 0.40 |
| FUSIDICACID-50 | TOBRAMYCIN-0.4 | -0.04 | 0.46 |
| TRIMETHOPRIM-0.2 | VANCOMYCIN-50 | -0.14 | -1.31 |
| FUSIDICACID-50 | NITROFURANTOIN-2.0 | -0.18 | 1.18 |
| CEFOXITIN-1.0 | RIFAMPICIN-1.0 | -0.30 | 1.12 |
| CEFOXITIN-1.0 | SPECTINOMYCIN-4.0 | -0.31 | 2.31 |
| ERYTHROMYCIN-5.0 | RIFAMPICIN-1.0 | -0.32 | 0.13 |
| RIFAMPICIN-1.0 | TRIMETHOPRIM-0.2 | -0.32 | 0.40 |
| CLARYTHROMYCIN-5.0 | VANCOMYCIN-50 | -0.33 | -0.06 |
| SPECTINOMYCIN-4.0 | TRIMETHOPRIM-0.2 | -0.37 | 1.68 |
| ERYTHROMYCIN-5.0 | VANCOMYCIN-50 | -0.40 | 0.03 |
| CEFOXITIN-1.0 | VANCOMYCIN-50 | -0.41 | 0.83 |
| CEFOXITIN-1.0 | FUSIDICACID-50 | -0.41 | -0.77 |
| ERYTHROMYCIN-5.0 | SPECTINOMYCIN-4.0 | -0.43 | 0.23 |
| ERYTHROMYCIN-5.0 | FUSIDICACID-50 | -0.44 | -1.32 |
| AMIKACIN-0.2 | RIFAMPICIN-1.0 | -0.47 | 1.10 |
| AMIKACIN-0.2 | SPECTINOMYCIN-4.0 | -0.49 | 2.22 |
| GENTAMICIN-0.1 | SPECTINOMYCIN-4.0 | -0.50 | 2.69 |
| AMIKACIN-0.2 | VANCOMYCIN-50 | -0.50 | 1.06 |
| GENTAMICIN-0.1 | RIFAMPICIN-1.0 | -0.51 | 1.49 |
| GENTAMICIN-0.1 | VANCOMYCIN-50 | -0.51 | 0.88 |
| FUSIDICACID-50 | TRIMETHOPRIM-0.2 | -0.53 | 1.65 |
| SPECTINOMYCIN-4.0 | VANCOMYCIN-50 | -0.61 | 0.84 |
| CLARYTHROMYCIN-5.0 | RIFAMPICIN-1.0 | -0.62 | -0.33 |
| RIFAMPICIN-1.0 | TETRACYCLINE-1.0 | -0.67 | -0.25 |
| CLARYTHROMYCIN-5.0 | SPECTINOMYCIN-4.0 | -0.68 | 0.26 |
| SPECTINOMYCIN-4.0 | TETRACYCLINE-1.0 | -0.68 | 0.21 |
| OXACILLIN-40.0 | VANCOMYCIN-50 | -0.69 | -0.18 |
| RIFAMPICIN-1.0 | SPECTINOMYCIN-4.0 | -0.71 | 0.32 |
| OXACILLIN-40.0 | RIFAMPICIN-1.0 | -0.74 | 0.91 |
| AMIKACIN-0.2 | FUSIDICACID-50 | -0.74 | -0.55 |
| CLARYTHROMYCIN-5.0 | FUSIDICACID-50 | -0.78 | -1.37 |
| RIFAMPICIN-1.0 | VANCOMYCIN-50 | -0.78 | 0.79 |
| FUSIDICACID-50 | RIFAMPICIN-1.0 | -0.79 | -0.82 |
| TETRACYCLINE-1.0 | VANCOMYCIN-50 | -0.82 | -1.02 |
| FUSIDICACID-50 | GENTAMICIN-0.1 | -0.84 | 0.70 |
| FUSIDICACID-50 | VANCOMYCIN-50 | -0.85 | -1.39 |
| OXACILLIN-40.0 | SPECTINOMYCIN-4.0 | -0.92 | 0.14 |
| FUSIDICACID-50 | SPECTINOMYCIN-4.0 | -0.94 | 0.43 |
| FUSIDICACID-50 | OXACILLIN-40.0 | -0.96 | -1.31 |
| FUSIDICACID-50 | TETRACYCLINE-1.0 | -1.04 | -0.52 |

Appendix Table S1: Predicted and measured interaction score for new drug interaction pairs in the test set by INDIGO. Interactions were z-transformed and ordered based on most antagonistic to most synergistic based on predictions by INDIGO. The drug name along with the dose used in the chemogenomics data is shown.

| Antibiotic Name | MIC (ug) |
| --- | --- |
| Cefoxitin | 2 |
| Clarithromycin | 0.5 |
| Erythromycin | 0.3 |
| Gentamicin | 1.8 |
| Nitrofurantoin | 20 |
| Oxacillin | 12 |
| Rifampicin | 4 |
| Tetracycline | 1.5 |

## Appendix Table S2: Antibiotics used and their MIC in *S. aureus*

| Drug | Chosen for testing? | Minimum Deviation score | Max. Deviation score | Range |
| --- | --- | --- | --- | --- |
| CLARYTHROMYCIN | Yes | -2.33 | 1.62 | 3.95 |
| VANCOMYCIN | Yes | -2.35 | 1.42 | 3.77 |
| CHLORAMPHENICOL | Yes | -1.94 | 1.80 | 3.74 |
| TETRACYCLINE | Yes | -2.00 | 1.72 | 3.73 |
| RIFAMPICIN | Yes | -1.94 | 1.77 | 3.72 |
| ERYTHROMYCIN | Yes | -2.34 | 1.36 | 3.70 |
| TRIMETHOPRIM |  | -1.86 | 1.77 | 3.64 |
| LEVOFLOXACIN |  | -1.57 | 1.80 | 3.36 |
| SPECTINOMYCIN |  | -1.41 | 1.90 | 3.30 |
| FUSIDICACID |  | -2.35 | 0.89 | 3.24 |
| OXACILLIN | Yes | -2.16 | 1.05 | 3.21 |
| CEFOXITIN | Yes | -1.86 | 1.22 | 3.08 |
| NALIDIXICACID |  | -1.57 | 1.51 | 3.07 |
| CIPROFLOXACIN |  | -1.34 | 1.72 | 3.07 |
| NITROFURANTOIN | Yes | -1.38 | 1.67 | 3.05 |
| TOBRAMYCIN |  | -1.13 | 1.90 | 3.03 |
| AMIKACIN |  | -1.44 | 0.98 | 2.42 |
| GENTAMICIN | Yes | -1.31 | 1.04 | 2.35 |
| PEROXIDE |  | 0.78 | 1.77 | 1.00 |

Appendix Table S3: Prioritizing interactions for testing in S. aureus**.** Drugs were prioritized based on degree of deviation predicted by INDIGO; in addition, drugs representative of different classes were chosen for experimental testing.

| Drug 1 | Drug 2 | S. aureus experimental interaction score | E. coli experimental interaction score | Predicted Deviation Score by INDIGO (E. coli - S. aureus) |
| --- | --- | --- | --- | --- |
| CEFOXITIN | CHLORAMPHENICOL | 0.9235 | 0.769 | -0.56602 |
| CEFOXITIN | CLARYTHROMYCIN | 0.3065 | 0.1665 | -0.96696 |
| CEFOXITIN | ERYTHROMYCIN | 1.378 | 0.0975 | -0.94549 |
| CEFOXITIN | GENTAMICIN | 1.2775 | 0.2985 | -0.63685 |
| CEFOXITIN | NITROFURANTOIN | 0.5925 | 2.886 | 0.97325 |
| CEFOXITIN | OXACILLIN | -0.227 | -0.329 | -1.16468 |
| CEFOXITIN | RIFAMPICIN | 0.836 | 1.121 | -0.31834 |
| CEFOXITIN | TETRACYCLINE | -0.0135 | -0.0315 | -1.29517 |
| CEFOXITIN | VANCOMYCIN | 1.35 | 0.834 | -0.58009 |
| CHLORAMPHENICOL | CLARYTHROMYCIN | -0.7675 | -0.722 | -1.58725 |
| CHLORAMPHENICOL | ERYTHROMYCIN | -0.0535 | -0.7355 | -1.54307 |
| CHLORAMPHENICOL | GENTAMICIN | -0.262 | 1.939 | 0.76576 |
| CHLORAMPHENICOL | NITROFURANTOIN | 0.567 | 1.2535 | 0.41761 |
| CHLORAMPHENICOL | OXACILLIN | 1.2455 | 1.213 | -0.34186 |
| CHLORAMPHENICOL | RIFAMPICIN | 1.2325 | 0.244 | -0.89769 |
| CHLORAMPHENICOL | TETRACYCLINE | 0.4995 | -0.6835 | -1.58016 |
| CHLORAMPHENICOL | VANCOMYCIN | 0.8485 | 0.081 | -0.99278 |
| CLARYTHROMYCIN | ERYTHROMYCIN | 1.182 | -0.173 | -1.30134 |
| CLARYTHROMYCIN | GENTAMICIN | 1.346 | 0.442 | -0.72614 |
| CLARYTHROMYCIN | NITROFURANTOIN | -0.0105 | 0.1915 | -0.68263 |
| CLARYTHROMYCIN | OXACILLIN | 0.3725 | 0.767 | -0.61688 |
| CLARYTHROMYCIN | RIFAMPICIN | 0.7525 | -0.3265 | -1.24528 |
| CLARYTHROMYCIN | TETRACYCLINE | -1.0875 | -0.9095 | -1.91199 |
| CLARYTHROMYCIN | VANCOMYCIN | 0.7485 | -0.065 | -1.16841 |
| ERYTHROMYCIN | GENTAMICIN | 0.131 | 0.422 | -0.70405 |
| ERYTHROMYCIN | NITROFURANTOIN | -0.293 | 0.593 | -0.35944 |
| ERYTHROMYCIN | OXACILLIN | 0.5685 | 1.134 | -0.42438 |
| ERYTHROMYCIN | RIFAMPICIN | 1.3585 | 0.134 | -1.06548 |
| ERYTHROMYCIN | TETRACYCLINE | -1.4045 | -0.787 | -1.83516 |
| ERYTHROMYCIN | VANCOMYCIN | 1.5845 | 0.026 | -1.10246 |
| GENTAMICIN | NITROFURANTOIN | 0.813 | 0.017 | -1.1152 |
| GENTAMICIN | OXACILLIN | 3.2085 | 0.0385 | -1.02681 |
| GENTAMICIN | RIFAMPICIN | 1.3835 | 1.4905 | 0.03037 |
| GENTAMICIN | TETRACYCLINE | 0.123 | -0.6605 | -1.12231 |
| GENTAMICIN | VANCOMYCIN | 0.6055 | 0.88 | -0.38625 |
| NITROFURANTOIN | OXACILLIN | 3.7195 | 3.345 | 1.04752 |
| NITROFURANTOIN | RIFAMPICIN | 1.6025 | 3.022 | 1.05094 |
| NITROFURANTOIN | TETRACYCLINE | 0.801 | 1.607 | 0.33958 |
| NITROFURANTOIN | VANCOMYCIN | 2.005 | 0.397 | -0.71846 |
| OXACILLIN | RIFAMPICIN | 1.446 | 0.909 | -0.54285 |
| OXACILLIN | TETRACYCLINE | 1.6625 | -0.7935 | -1.76672 |
| OXACILLIN | VANCOMYCIN | 0.0795 | -0.183 | -1.11027 |
| RIFAMPICIN | TETRACYCLINE | 0.214 | -0.252 | -1.37056 |
| RIFAMPICIN | VANCOMYCIN | 1.7955 | 0.79 | -0.57059 |
| TETRACYCLINE | VANCOMYCIN | 0.398 | -1.018 | -1.90355 |

Appendix Table S4: Drug Interaction scores in S. aureus. Experimentally measured interaction scores for drug pairs in S. aureus are shown in columns 1 and the corresponding values for E. coli are shown in column 2. To identify interactions that would potentially differ between the two species, we deleted the scores of non-orthologous genes in the trained E. coli INDIGO model and identified drug interactions that are most sensitive to this alteration. The amount of difference between the original interaction score and the modified interaction score is shown in column 3.

| Drug 1 | Drug 2 | *M. tuberculosis* interaction score | Predicted interaction score for *E.coli* | Predicted deviation score |
| --- | --- | --- | --- | --- |
| SPECTINOMYCIN | MITOMYCINC | 1.00 | 1.39 | -0.39 |
| SPECTINOMYCIN | CLARYTHROMYCIN | -1.00 | -1.01 | -1.95 |
| SPECTINOMYCIN | FUSIDICACID | -1.00 | -2.12 | -2.67 |
| SPECTINOMYCIN | AZITHROMYCIN | -1.00 | -0.76 | -1.76 |
| SPECTINOMYCIN | RIFAMPICIN | -1.00 | -0.52 | -1.65 |
| SPECTINOMYCIN | TETRACYCLINE | -1.00 | -1.64 | -2.39 |
| SPECTINOMYCIN | VANCOMYCIN | 1.00 | -0.55 | -1.60 |
| SPECTINOMYCIN | ISONIAZID | 0.00 | 1.14 | -0.51 |
| SPECTINOMYCIN | STREPTOMYCIN | 1.00 | 1.44 | -0.33 |
| SPECTINOMYCIN | MINOCYCLINE | -1.00 | -0.61 | -1.70 |
| SPECTINOMYCIN | NOVOBIOCIN | -1.00 | -0.59 | -1.68 |
| RIFAMPICIN | ISONIAZID | 0.00 | 0.86 | -0.76 |
| RIFAMPICIN | CLARYTHROMYCIN | 0.00 | -1.10 | -2.01 |
| RIFAMPICIN | MINOCYCLINE | 0.00 | -0.54 | -1.67 |
| RIFAMPICIN | STREPTOMYCIN | 0.00 | 1.40 | -0.36 |
| ISONIAZID | CLARYTHROMYCIN | 0.00 | 0.35 | -1.08 |
| ISONIAZID | MINOCYCLINE | 0.00 | 0.82 | -0.79 |
| ISONIAZID | STREPTOMYCIN | 0.00 | 0.23 | -1.18 |
| SPECTINOMYCIN | NIGERICIN | -1.00 | 1.13 | -0.52 |
| CYCLOSERINED | RIFAMPICIN | 0.00 | 0.96 | -0.62 |
| CYCLOSERINED | STREPTOMYCIN | 0.00 | 0.35 | -1.03 |
| CYCLOSERINED | AMIKACIN | 0.00 | -0.26 | -1.39 |
| CYCLOSERINED | ISONIAZID | 0.00 | 0.04 | -1.28 |
| ISONIAZID | AMIKACIN | 0.00 | -0.39 | -1.55 |

Appendix Table S5: Drug Interaction scores in M. tuberculosis. Experimentally measured interaction scores for drug pairs in *M. tuberculosis* from literature are shown in columns 1 and the corresponding values for *E. coli* predicted by INDIGO are shown in column 2. The predicted difference between the *E.coli* interaction score and the *M. tuberculosis* interaction score is shown in column 3.

| **Drug** | **Closest Chemogenomics Dose** | **Modified Chemogenomics Dose** |
| --- | --- | --- |
| Cef | Cefoxitin-1.0 | Cefoxitin-1.0* |
| Chl | Chloramphenicol-1.5 | Chloramphenicol-0.5 |
| Cip | Ciprofloxacin-0.004 | Ciprofloxacin-0.008 |
| Cla | Clarythromycin-5.0 | Clarythromycin-0.1 |
| Ery | Erythromycin-5.0 | Erythromycin-0.1 |
| Fus | Fusidicacid-50 | Fusidicacid-1 |
| Gen | Gentamicin-0.1 | Gentamicin-0.05 |
| H22 | Peroxide-2.0 | Peroxide-0.1 |
| Lev | Levofloxacin-0.002 | Levofloxacin-0.002* |
| Nal | Nalidixicacid-2.0 | Nalidixicacid-0.5 |
| Nit | Nitrofurantoin-2.0 | Nitrofurantoin-0.1 |
| Oxa | Oxacillin-40.0 | Oxacillin-0.5 |
| Rif | Rifampicin-1.0 | Rifampicin-2.0 |
| Tet | Tetracycline-1.0 | Tetracycline-0.25 |
| Tri | Trimethoprim-0.2 | Trimethoprim-0.4 |
| Van | Vancomycin-50 | Vancomycin-10 |
| Tob | Tobramycin-0.4 | Tobramycin-0.05 |
| Amk | Amikacin-0.2 | Amikacin-0.05 |
| Spe | Spectinomycin-4.0 | Spectinomycin-6.0 |

Appendix Table S6: Effect of Dose of Chemogenomics Profiles on INDIGO Predictions. The dose used in chemogenomics data also influences drug interaction predictions. For training and testing INDIGO, we matched the closest dose in chemogenomics data to the experimentally measured dosage (shown in column 2). We found that choosing a dose in chemogenomics data that’s significantly different from the dose range in drug interaction data (shown in column 3), reduced the accuracy in test set and cross validation (Pearson’s correlation, R = 0.42 for test set and R = 0.39 for leave one drug out cross validation). Using the best matching dose the accuracy was R = 0.57 for test set R = 0.56 for cross validation. This analysis was done for those drugs for which multiple dose existed; profiles for Cef and Lev were available at only one dose in the chemogenomics data. This analysis suggests that the predictive ability of the model can be improved by choosing the dose for chemogenomics data that is closest to the experimentally measured dose for interaction, or by determining the interaction outcomes at multiple doses.

# References:

Brynildsen MP, Winkler JA, Spina CS, MacDonald IC, Collins JJ (2013) Potentiating antibacterial activity by predictably enhancing endogenous microbial ROS production. *Nature biotechnology* **31:** 160-165

Dwyer DJ, Belenky PA, Yang JH, MacDonald IC, Martell JD, Takahashi N, Chan CT, Lobritz MA, Braff D, Schwarz EG, Ye JD, Pati M, Vercruysse M, Ralifo PS, Allison KR, Khalil AS, Ting AY, Walker GC, Collins JJ (2014) Antibiotics induce redox-related physiological alterations as part of their lethality. *Proceedings of the National Academy of Sciences of the United States of America* **111:** E2100-2109

Imlay JA (2015) Diagnosing oxidative stress in bacteria: not as easy as you might think. *Current opinion in microbiology* **24:** 124-131

Lobritz MA, Belenky P, Porter CBM, Gutierrez A, Yang JH, Schwarz EG, Dwyer DJ, Khalil AS, Collins JJ (2015) Antibiotic efficacy is linked to bacterial cellular respiration. *Proceedings of the National Academy of Sciences*

Nichols RJ, Sen S, Choo YJ, Beltrao P, Zietek M, Chaba R, Lee S, Kazmierczak KM, Lee KJ, Wong A, Shales M, Lovett S, Winkler ME, Krogan NJ, Typas A, Gross CA (2011) Phenotypic landscape of a bacterial cell. *Cell* **144:** 143-156

Ocampo PS, Lázár V, Papp B, Arnoldini M, zur Wiesch PA, Busa-Fekete R, Fekete G, Pál C, Ackermann M, Bonhoeffer S (2014) Antagonism between bacteriostatic and bactericidal antibiotics is prevalent. *Antimicrobial agents and chemotherapy* **58:** 4573-4582

Qi Y (2012) Random forest for bioinformatics. In *Ensemble machine learning*, pp 307-323. Springer

Strobl C, Boulesteix A-L, Zeileis A, Hothorn T (2007) Bias in random forest variable importance measures: Illustrations, sources and a solution. *BMC Bioinformatics* **8:** 25

Yeh P, Tschumi AI, Kishony R (2006) Functional classification of drugs by properties of their pairwise interactions. *Nature genetics* **38:** 489-494
