# Supplementary material for: Chemogenomics and orthology‐based design of antibiotic combination therapies
Source: Mol Syst Biol. 2016 May 23;12(5):872. doi: 10.15252/msb.20156777 (PMC5289223; doi:10.15252/msb.20156777)
Supplement: Supplementary file 2 — Expanded View Figures PDF [file MSB-12-872-s002.pdf]

Expanded View Figures

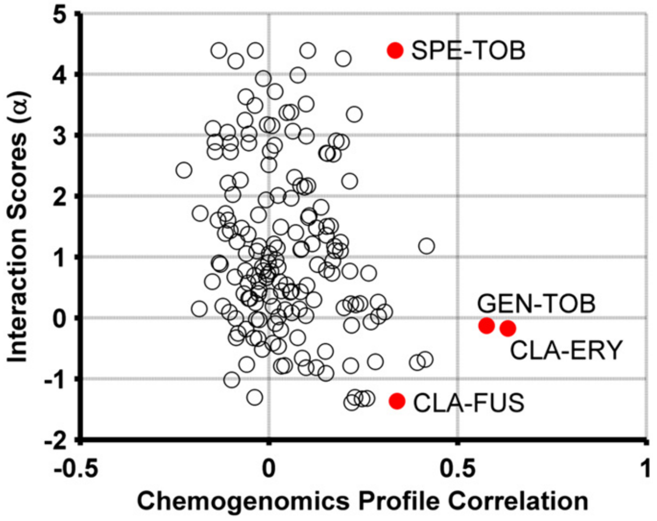

Figure EV1. Drugs with similar targets and chemogenomic profiles can have both synergistic and antagonistic interactions.
Combinations highlighted in this plot tobramycin–spectinomycin, tobramycin–gentamicin, and fusidic acid–clarithromycin share similar chemogenomic profiles, as measured by the correlation between their chemogenomic profiles, and similar target processes. Yet they have antagonistic, neutral, or synergistic outcomes, suggesting that chemogenomic profile or target similarity is not a strong predictor of drug-interaction outcome.

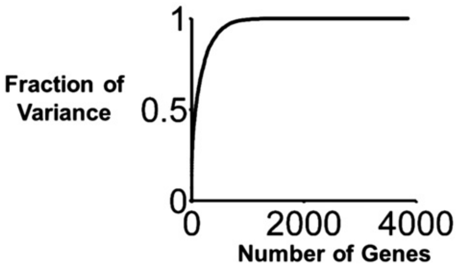

Figure EV2. Genes that are predictive of drug interactions in the INDIGO model.
The random forest algorithm was used to prioritize the genes based on their relative contribution in predicting drug interactions. The plot shows the fraction of variance in the interaction data predicted by the prioritized list of genes. We found that the top 81 genes accounted for 50%, the top 222 accounted for 75%, and the top 581 accounted for 95% of variance in the predicted data.

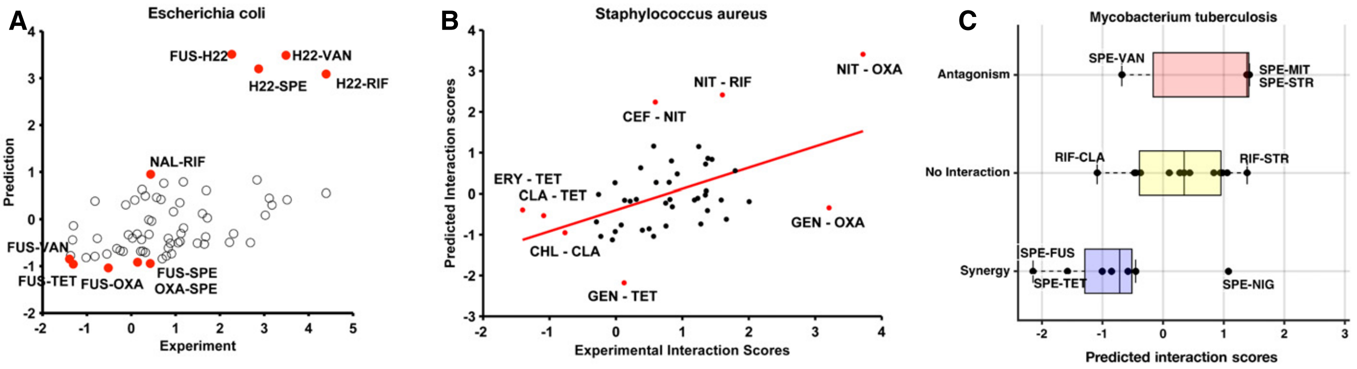

Figure EV3. Scatter plots of measured interaction scores and predictions by INDIGO with drug combination labels (raw scores).

A–C We have used rank-normalized data for visualization in Figs 3 and 5, and for quantifying the accuracy of predictions as they are robust to outliers and the method of normalization. The correlations are higher with actual values (Pearson's correlation R = 0.57, P-value = 10^{-7}) compared to rank-transformed values (R = 0.52, P-value = 10^{-6}) for *Escherichia coli* predictions. Strong synergistic, antagonistic interactions and outliers are highlighted for *E. coli* (panel A), *Staphylococcus aureus* (panel B; rank correlation R = 0.47; Pearson's correlation R = 0.5), and *Mycobacterium tuberculosis* (panel C; rank & Pearson's correlation R = 0.54) (*M. tuberculosis* interaction data are qualitative in nature). NIG—nigericin, STR—streptomycin, CLA—clarithromycin, MIT—mitomycin. Please refer to Table 1 for full list of drugs and abbreviations.

 © 2016 The Authors

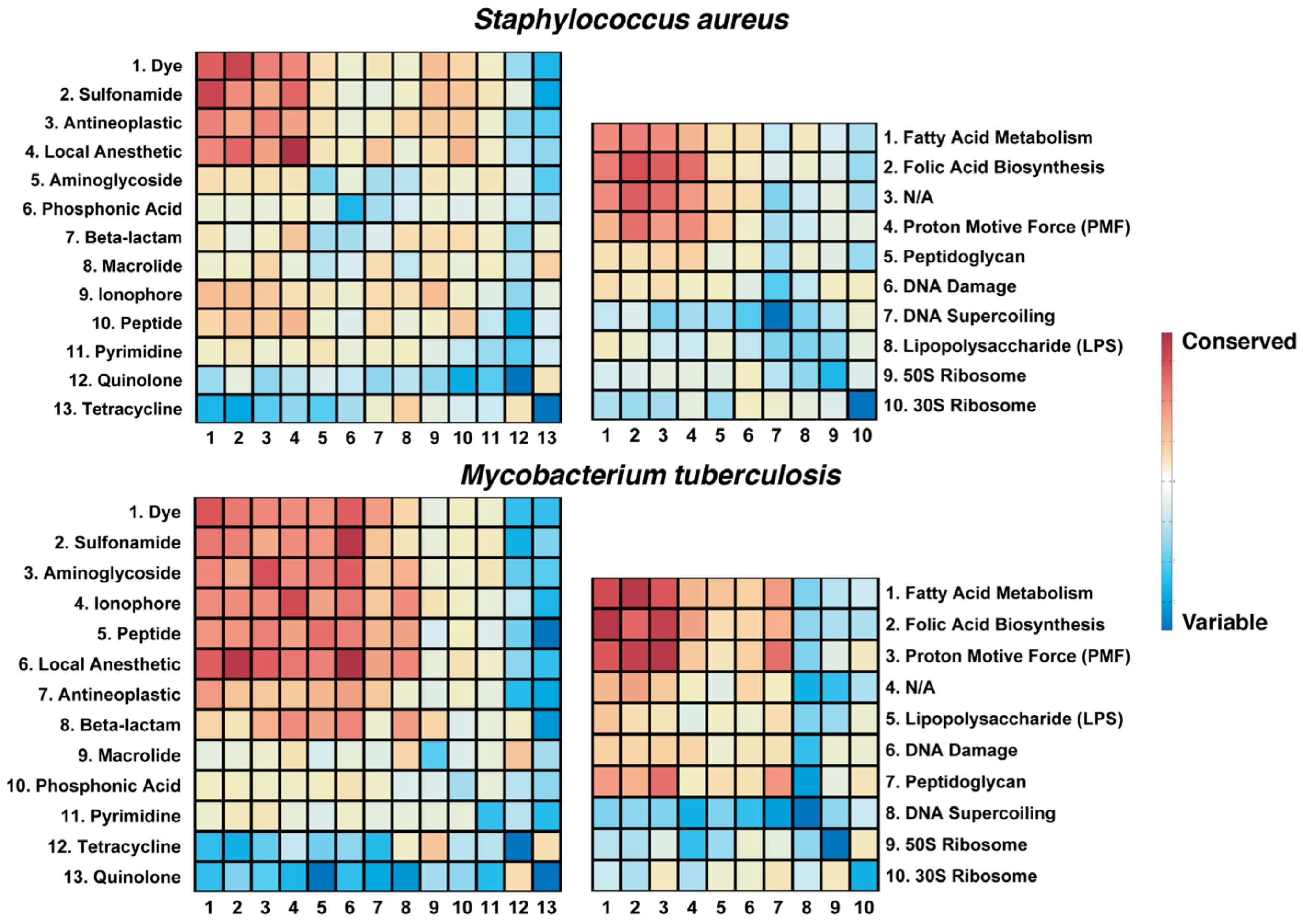

Figure EV4. Interaction conservation between *Escherichia coli* and *Staphylococcus aureus* (top panel) and *E. coli* and *Mycobacterium tuberculosis* (bottom panel).

The heat maps summarize the interaction outcome between drugs from similar or different chemical families (left panel) and target processes (right panel). The heat maps represent the entire drug-interaction matrix, inferred from the compendium of Nichols *et al*, collapsed into 13 major drug families and 10 major target processes. To determine the average conservation of interactions between different groups, we compared the differences in interaction scores between the two species for all drug combinations between two groups (drug family or target process) with the background interaction score difference for all drug pairs.
