# Supplementary material for: Chemogenomics and orthology‐based design of antibiotic combination therapies
Source: Mol Syst Biol. 2016 May 23;12(5):872. doi: 10.15252/msb.20156777 (PMC5289223; doi:10.15252/msb.20156777)
Supplement: Supplementary file 6 — Code EV1 [file MSB-12-872-s006.zip › INDIGO_DATA/INDIGO_README.docx]

**Instructions for using INDIGO**

**Installation:**

Make sure all the provided files are in the same folder or added to the MATLAB path. You also need the Random Forest toolbox for MATLAB -

<https://code.google.com/p/randomforest-matlab/downloads/list>

Download and extract files to the MATLAB folder and add to MATLAB path. You are now ready to run INDIGO.

**Examples:**

INDIGO takes in drug interaction scores and chemogenomic data to train the model. The trained model can be used to predict new drug interactions.

The INDIGO implementation is simple and very easy to run; here is an example -

>> [train_interactions, train_interaction_scores, phenotype_labels, indigo_model] = indigo_train('ecoli_training.xlsx','identifiers_match.xlsx','ecoli_phenotype_data_cell.xlsx');

The excel files that are used as input are provided with the INDIGO code.

Description of input files:

ecoli_training.xlsx – has the experimental drug interaction data (training set)

ecoli_phenotype_data_cell.xlsx – has the chemogenomic data taken from the supplementary information of Nichols et al, 2011.

identifiers_match.xlsx – It matches the drug identifiers used in the drug interaction data with the chemogenomic data.

Output:

The command outputs the trained model (indigo_model)

train_interactions, train_interaction_scores – these two variables have the interaction data used as input along with the drug names

phenotype_labels – has the gene identifiers in the chemogenomic data

The trained model (indigo_model) can be used to predict new drug interactions –

>> [test_interactions, testinteractions_scores] = indigo_predict(indigo_model,{VAN},1,'identifiers_match.xlsx','ecoli_phenotype_data_cell.xlsx');

This command (indigo_predict) takes in the trained model along with the drug of interest (VAN – vancomycin) for which interaction has to be inferred.

It also requires the list of drug identifiers for matching the drug interaction data with the chemogenomic data. Note that if the names of the drugs in the interaction data matches the chemogenomic data, the contents of the file can be left blank.

We can also simultaneously predict interactions for multiple drugs -

>> testdrugs = {'VAN';'SPE'; 'FUS';'RIF'};

>> [test_interactions, testinteractions_scores] = indigo_predict(indigo_model,testdrugs,1,'identifiers_match.xlsx','ecoli_phenotype_data_cell.xlsx');

Output:

test_interactions – This has the list of interactions predicted by INDIGO. The identifiers in the chemogenomic data is used to identify the drugs. Interactions are ordered alphabetically.

test_interactions_scores – This has the interaction scores for the drug interactions predicted by INDIGO. Interactions are ordered alphabetically (same order as test_interactions).

**Comparing predictions with experiment:**

First load the experimental drug interaction data for the test set drugs (provided with the code)

>> [test_exp_interaction_scores, test_exp_interaction_pairs] = xlsread('ecoli_test.xlsx');

Find the correlation with experimental data -

>> corr2(test_exp_interaction_scores,testinteractions_scores)

This should output ~ 0.55, the correlation between predicted and experimental data reported in Figure 2 of the manuscript.

INDIGO can also be used to predict specific interactions instead of all interactions for a drug. The parameter ‘input_type’ can be set to 2 (for interaction prediction) or 1 (for a specific drug).

>> testdrugs = {'VAN','TOB';'NIT','OXA';'CHL','OXA';'AMK','CEF';'FUS','TOB'}; input_type = 2;

>> [test_interactions, testinteractions_scores] = indigo_predict(indigo_model,testdrugs,input_type,'identifiers_match.xlsx','ecoli_phenotype_data_cell.xlsx');

Instead of inputting the data as an excel file, INIDIGO can also directly take in input data (chemogenomics or drug interaction data) as MATLAB arrays

>> [phenotype_data, phenotype_labels, conditions] = process_chemgen('ecoli_phenotype_data_cell.xlsx', z);

>> [interaction_scores, interaction_pairs] = xlsread('ecoli_training.xlsx');

>> [train_interactions, train_xn_scores, phenotype_labels, indigo_model] = indigo_train([] , 'identifiers_match.xlsx', [] ,2, phenotype_data, phenotype_labels, conditions, interaction_scores, interaction_pairs);

**Orthology based predictions:**

Train INDIGO on the entire drug interaction data and get E. coli model

>> [interaction_list, interaction_scores, labels, indigo_model, sigma_delta_scores, conditions] = indigo_train('ecoli_fulldata.xlsx','identifiers_match.xlsx','ecoli_phenotype_data_cell.xlsx');

The model (indigo_model) is now trained on the entire dataset (both training and test data). The excel file ecoli_fulldata.xlsx has the entire drug interaction dataset. The interactions are stored in the variable ‘interaction_scores’.

Output:

In addition to the output variables described earlier, this command also outputs –

conditions – The list of drugs or conditions for which chemogenomic data is available

sigma_delta_scores – This has the sigma and delta scores calculated by INDIGO for each drug interaction pair.

Now load the list of orthologs between *E. coli* and *S. aureus* -

>> [num,ecoli_staph_orth] = xlsread('ecoli_staph_orthologs.xlsx');

The excel file ecoli_staph_orthologs.xlsx is provided with the code.

Now use INDIGO to estimate the interactions that would differ between the two species

>> deviations = indigo_orthology(labels, ecoli_staph_orth, sigma_delta_scores, indigo_model);

Inputs:

labels - gene identifiers in the chemogenomic data (output of indigo_train)

ecoli_staph_orth – orthologs between *E. coli* and *S. aureus*

sigma_delta_scores - sigma and delta scores calculated by INDIGO for each drug interaction pair (output of indigo_train)

indigo_model – INDIGO model trained on the entire dataset (output of indigo_train)

Output:

deviations – Predicted difference in interaction score between *E. coli* and *S. aureus*. Interactions are in the same order as in the variable ‘interaction_list’
